# MG132 exerts anti-viral activity against HSV-1 by overcoming virus-mediated suppression of the ERK signaling pathway

**DOI:** 10.1038/s41598-020-63438-1

**Published:** 2020-04-21

**Authors:** Hanako Ishimaru, Kohei Hosokawa, Atsuko Sugimoto, Riho Tanaka, Tadashi Watanabe, Masahiro Fujimuro

**Affiliations:** Department of Cell Biology, Kyoto Pharmaceutical University, Misasagi-Shichonocho 1, Yamashinaku, Kyoto 607-8412 Japan

**Keywords:** Proteasome, Growth factor signalling, Ubiquitylation, Hepatitis B, Viral infection

## Abstract

Herpes simplex virus 1 (HSV-1) causes a number of clinical manifestations including cold sores, keratitis, meningitis and encephalitis. Although current drugs are available to treat HSV-1 infection, they can cause side effects such as nephrotoxicity. Moreover, owing to the emergence of drug-resistant HSV-1 strains, new anti-HSV-1 compounds are needed. Because many viruses exploit cellular host proteases and encode their own viral proteases for survival, we investigated the inhibitory effects of a panel of protease inhibitors (TLCK, TPCK, E64, bortezomib, or MG132) on HSV-1 replication and several host cell signaling pathways. We found that HSV-1 infection suppressed c-Raf-MEK1/2-ERK1/2-p90RSK signaling in host cells, which facilitated viral replication. The mechanism by which HSV-1 inhibited ERK signaling was mediated through the polyubiquitination and proteasomal degradation of Ras-guanine nucleotide-releasing factor 2 (Ras-GRF2). Importantly, the proteasome inhibitor MG132 inhibited HSV-1 replication by reversing ERK suppression in infected cells, inhibiting lytic genes (ICP5, ICP27 and UL42) expression, and overcoming the downregulation of Ras-GRF2. These results indicate that the suppression of ERK signaling via proteasomal degradation of Ras-GRF2 is necessary for HSV-1 infection and replication. Given that ERK activation by MG132 exhibits anti-HSV-1 activity, these results suggest that the proteasome inhibitor could serve as a novel therapeutic agent against HSV-1 infection.

## Introduction

Herpes simplex virus 1 (HSV-1) is a member of the *Alphaherpesvirinae* subfamily and a human DNA virus that is known to cause a number of clinical manifestations, including cold sores, keratitis, meningitis and encephalitis^1,2^. HSV-1 can establish latent infections in sensory neurons and periodically reactivate at the original site of infection, resulting in lesions^3^. During latent infection, the HSV genome circularizes to form an episome in the nucleus, leading to expression of latency-associated transcripts (LATs)  that are thought to be necessary for latency and reactivation. Upon reactivation, lytic-related genes are expressed in a temporal and sequential manner, which can be divided into three transcriptional stages: immediate early (IE/α), early (E/β), and late (L/γ). Some IE products function as triggers for transcriptional activation of E genes associated with viral DNA replication. L genes encode structural and functional proteins for producing viral progeny.

Although acyclovir (ACV) and its analogues have been the standard therapy for HSV infection, their widespread and long-term use has recently led to the emergence of drug-resistant HSV strains^4–6^. Thus, due to a lack of effective vaccines, side effects associated with ACV, such as nephrotoxicity, and appearance of ACV-resistant strains, new anti-HSV compounds with mechanisms of inhibition distinct from ACV are urgently needed for the treatment of HSV infection^7^.

HSV infection alters several signaling pathways, which can be triggered by viral molecules known as pathogen associated molecular patterns (PAMPs). PAMPs are detected by sentinel receptors such as toll-like receptors (TLRs) and induce the activation of NF-κB and IRF for initiating innate immune responses^8–12^. PAMPs derived from HSV can be detected by multiple TLRs in an infected cell or a dendritic cell^13,14^. NF-κB, is a major signaling pathway activated by HSV infection. In addition, the ERK and AKT signaling pathways are either dysregulated or utilized by tegument proteins or lytic proteins from a number of viruses including HSV, to establish infection, stimulate their replication, and suppress apoptosis^15–18^. Conflicting effects of HSV-1 infection on ERK suppression^19–21^ and activation have been reported^22–24^.

Cellular proteases play a key role in not only protein degradation but also in the regulation of signaling pathways, endocytosis, apoptosis, immune responses, and viral replication. Viruses exploit cellular proteases and encode their own viral proteases for survival, escape from immune responses, replication, assembly, entry and release^25,26^. In fact, several inhibitors of the aspartyl protease of HIV-1 and NS3/4A serine protease of hepatitis C virus have been approved for clinical use^6,27^. It has also been reported that HIV-protease inhibitors suppressed the replication of Kaposi sarcoma-associated herpesvirus (KSHV) and Epstein-Barr virus^28^, and proteasome inhibitors suppressed the replication of varicella zoster virus^29^, cytomegalovirus^30,31^, KSHV^32^, and HSV-1^33,34^.

Given the growing evidence supporting the importance of proteases in a physiological context, we hypothesized that protease inhibitors could be novel compounds for the treatment of HSV-1. We therefore investigated the inhibitory effects of several protease inhibitors on HSV replication and elucidated their underlying mechanisms.

## Results

### The proteasome inhibitor MG132 suppresses HSV-1 lytic gene expression and replication

**Figure 1:**
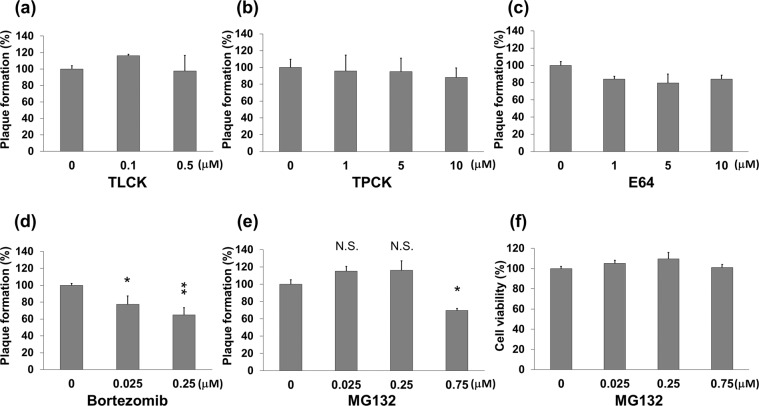
The inhibitory effects of TLCK, TPCK, E64, bortezomib, and MG132 on HSV-1 replication. (**a**–**e**) The bar graphs in a, b, c, d, and e show the anti-HSV-1 activities of TLCK, TPCK, E64, bortezomib, and MG132, respectively, calculated by plaque reduction assay. Vero cells cultured in 12-well tissue culture plate were infected with HSV-1 (HF strain) at 50 plaque forming units (PFU)/well for 30 min, and then cultured for 2 days with DMEM containing methylcellulose and serially diluted test compounds. Cell sheets were stained and fixed with crystal violet in 50% methanol, and the total number of plaques was quantified. The plaque number in vehicle (DMSO) treated cells was defined as 100%. Standard deviations were determined by analysis of data from three independent experiments, and are indicated by error bars. ^*^P < 0.05 and ^**^P < 0.01 indicate statistical significance compared with vehicle (DMSO) treated cells. N.S. indicates not statistically significant. (**f**) Cytotoxic effect of MG132 on Vero cells. Cells were incubated with various concentrations of MG132 for 48 hours and subjected to cell viability assays. The value of DMSO treated cells was defined as 100%.

By a plaque reduction assay, we investigated whether the protease inhibitors, tosyllysine chloromethyl ketone (TLCK), tosylphenylalanyl chloromethyl ketone (TPCK), E64, bortezomib, or MG132 could suppress HSV-1 replication. TLCK and TPCK inhibit the trypsin-like and chymotrypsin-like serine proteases, respectively. E64 is a cysteine protease inhibitor against calpain and caspases, and bortezomib and MG132 are proteasome inhibitors. Vero cells were incubated with HSV-1 for 30 min, cultured with medium containing various concentrations of each inhibitor for 2 days, and the plaque numbers of the inhibitor treated and untreated cells were quantified in order to determine the inhibition of virus yields (Fig. 1a–e). Bortezomib decreased the plaque numbers in a concentration dependent manner (Fig. 1d). The plaque numbers increased by 10–20% in low concentrations (0.025 and 0.25 μM) of MG132 treated cells, however, decreased by approximately 35% at a higher concentration (0.75 μM) of MG132 treated cells compared with vehicle treated cells (Fig. 1e). E64 treatment decreased plaque numbers by 10–20% (Fig. 1c), whereas TLCK and TPCK had no effect on plaque formation (Fig. 1a,b). It is worth noting that even concentrations as high as 0.75 μM of MG132 did not interfere with cell viability (Fig. 1f).

**Figure 2:**
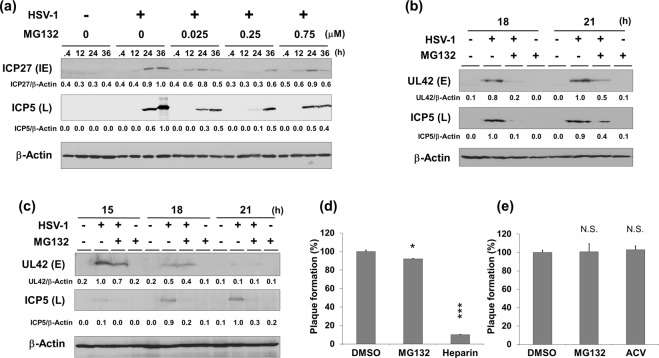
Effects of MG132 on HSV-1 lytic-protein expression, adsorption and entry. (**a**–**c**) Suppression of HSV-1 lytic protein expression by MG132 treatment. Vero (**a**), HepG2 (**b**) and H1299 (**c**) cells were mock infected or infected with HSV-1 at a multiplicity of infection (MOI) of 1 for 30 min, and then cultured with or without various concentrations of MG132 for 0.4, 12, 15, 18, 21, 24, or 36 hours. Cell lysates were subjected to Western blotting with anti-ICP5 (L/γ gene product), anti-UL42 (E/β gene product) and anti-ICP27 (IE/α gene product) antibodies. The band intensities of viral protein were calculated using ImageJ software and normalized to those of total β-Actin. The values of viral protein/β-actin are presented at the bottom of the image, and the highest value is presented as 1.0. Original images of blotting are shown in Supplementary Fig. S2. (**d**) The effect of MG132 on virus attachment to cells. Vero cells were incubated with 200 PFU HSV-1 with 0.75 μM MG132 or 0.01 unit/ml heparin for 30 min at 4 °C. Heparin inhibits HSV-1 attachment to host cells and was used as a positive control. The percentage of plaque number in DMSO treated cells was defined as 100%. (**e**) The effect of MG132 on HSV-1 penetration into cells. Vero cells were incubated with 100 PFU HSV-1 for 30 min at 4 °C, and DMEM containing virus was removed. Prewarmed medium containing 0.75 μM MG132 or 10 μM ACV was added, and cells were further incubated for 30 min at 37 °C. To remove virions from cell membrane, cells were washed with citrate buffer and PBS and cultured for 48 hours. The percentage of plaque number in DMSO treated cells was defined as 100%. (**d**,**e**) Standard deviations were determined by analyzing the data from three independent experiments and are indicated by the error bars. ^*^P < 0.05 and ^***^P < 0.001 indicate the statistical significance compared with vehicle-treated cells. N.S. indicates not statistically significant.

As proteasome inhibitors showed significant anti-HSV-1 activity, we focused on the inhibitory effect of MG132 on HSV-1 replication and examined the underlying molecular mechanisms. We next investigated the effects of MG132 treatment on lytic gene expression, virus adsorption and penetration (Fig. 2). Vero cells infected with HSV-1 were cultured with (or without) MG132 for 0.4–36 hours, and protein expression of ICP27 (IE gene) and ICP5 (L gene) were analyzed (Fig. 2a). Expression levels of ICP27 and ICP5 increased from 24 to 36 hours postinfection, however MG132 treatment decreased both ICP27 and ICP5 protein expression compared with no treatment. In addition to monkey-derived Vero cells, the effect of MG132 on lytic protein expression was also evaluated in human HepG2 (Fig. 2b) and H1299 cells (Fig. 2c). Although the expression of UL42 (E gene) and ICP5(L gene) increased up to 15 or 18 hours postinfection depending on the cell line, MG132 decreased the expression of both proteins. Furthermore, we investigated whether MG132 inhibited virus adsorption and entry into cells. A cell attachment and entry assay revealed that MG132 did not inhibit viral adsorption and entry of HSV-1 into Vero cells (Fig. 2d,e) while the positive control heparin inhibited absorption on the target cell.

**Figure 3:**
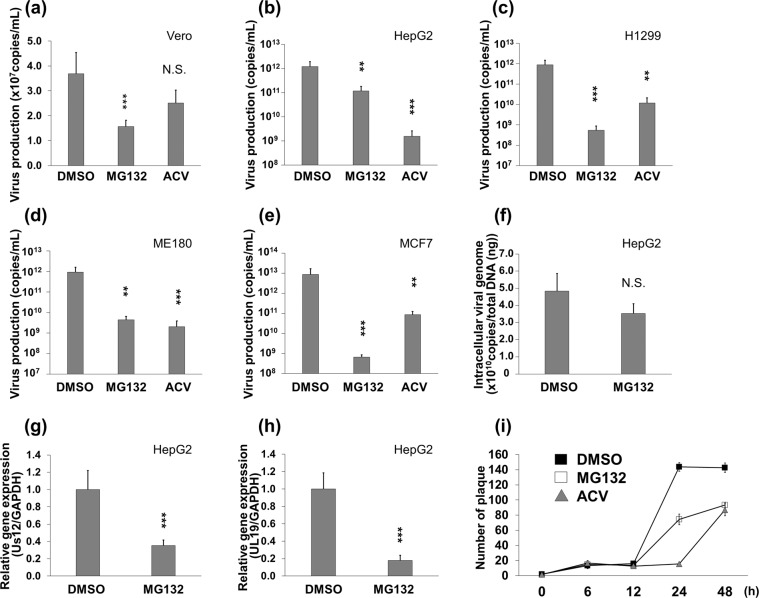
MG132 suppressed virus production, viral DNA replication and lytic genes expression. (**a**–**e**) Suppression of extracellular HSV-1 production by MG132. Vero (**a**), HepG2 (**b**), H1299 (**c**), ME180 (**d**) and MCF7 (**e**) were infected with HSV-1 at an MOI of 1 for 30 min. Cells were cultured with 0.75 μM MG132 (Vero and HepG2), 0.3 μM MG132 (H1299) or 0.25 μM MG132 (ME180 and MCF7) and 10 μM ACV for 24 hours, except for Vero cells. Vero cells were cultured with drug for 12 hours. The culture supernatants containing virus were harvested, and virus DNA were purified from culture supernatants and measured by qPCR. (**f**) The effect of MG132 on intracellular HSV-1 DNA replication. HepG2 cells were infected with HSV-1 at an MOI of 1 for 30 min and subsequently cultured with the drug for 43 hours. Viral DNA were isolated from cells and quantified by qPCR using UL19 specific primers. (**g**,**h**) Suppression of lytic genes transcription by MG132. HepG2 were infected with HSV-1 for 30 min and cultured with 0.75 μM MG132 for 20 hours, Total RNA was purified from cells and subjected to RT-qPCR to measure mRNA expression of Us12 (IE/α gene) and UL19 (L/γ gene). The mRNA expression of Us12 and UL19 were normalized by GAPDH mRNA expression. (**a**–**h**) Standard deviations were determined by analyzing the data from three experiments. ^**^P < 0.01 and ^***^P < 0.001 indicate the statistical significance compared with vehicle (DMSO) treated cells. N.S. indicates not statistically significant. (**i**) Analysis of viral replication cycle by one-step growth curve in the presence of MG132. HepG2 cells were infected with HSV-1 at 1 PFU/cell for 30 min and cultured with vehicle (DMSO), 0.75 μM MG132 or 10 μM ACV. The culture media was harvested at 0, 6, 12, 24 and 48 hours postinfection, and amount of virus in culture media was measured by a plaque reduction assay using Vero cells. The black square, white square and gray triangle indicate DMSO-, MG132- and ACV-treatment, respectively.

To gain further insight into the MG132-mediated anti-viral machinery, we analyzed the effects of MG132 on extracellular and intracellular HSV-1 production in human cells, in addition to a plaque reduction assay using Vero cells. Acyclovir (ACV) was used as a positive control inhibitor against viral DNA replication. Vero, HepG2, H1299, ME180 and MCF7 cells were infected with HSV-1 and subsequently cultured with MG132 for 24 hours. Viral genomic DNA was purified and extracted from enveloped virus in harvested culture supernatants and quantified by real-time PCR. As a result, MG132 treatment decreased extracellular HSV-1 production in culture supernatants from Vero cells by 41% (Fig. 3a). On the other hand, extracellular HSV-1 production in culture supernatants from HepG2 (Fig. 3b), H1299 (Fig. 3c), ME180 (Fig. 3d) and MCF7 (Fig. 3e) cells were significantly decreased to 10–0.01% by MG132. In addition to extracellular virus production, we also measured intracellular viral DNA and viral RNA expression in MG132-treated HepG2 cells by real-time and RT real-time PCR, respectively. The intracellular viral DNA replication in MG132-treated cells was reduced by about 20% (Fig. 3f), although there was no significant difference. The mRNA expression of IE gene Us12 and L gene UL19 in MG132-treated cells was decreased by about 70% and 80%, respectively (Fig. 3g,h). To test whether the kinetics of viral replication is slower in MG132-treated HepG2 cells relative to untreated cells, we compared replication kinetics with and without MG132 by a one-step growth curve (Fig. 3i). These data show that MG132 decreased the amount of infectious virions released from HepG2 cells at 24 and 48 hours postinfection. Taken together, our findings suggest that MG132 displays anti-HSV-1 activity by either suppressing lytic gene expression or influencing the early stage of lytic gene expression.

### MG132-treatment reverses HSV-1-mediated activation of NF-κB signaling by stabilizing IκBα

**Figure 4:**
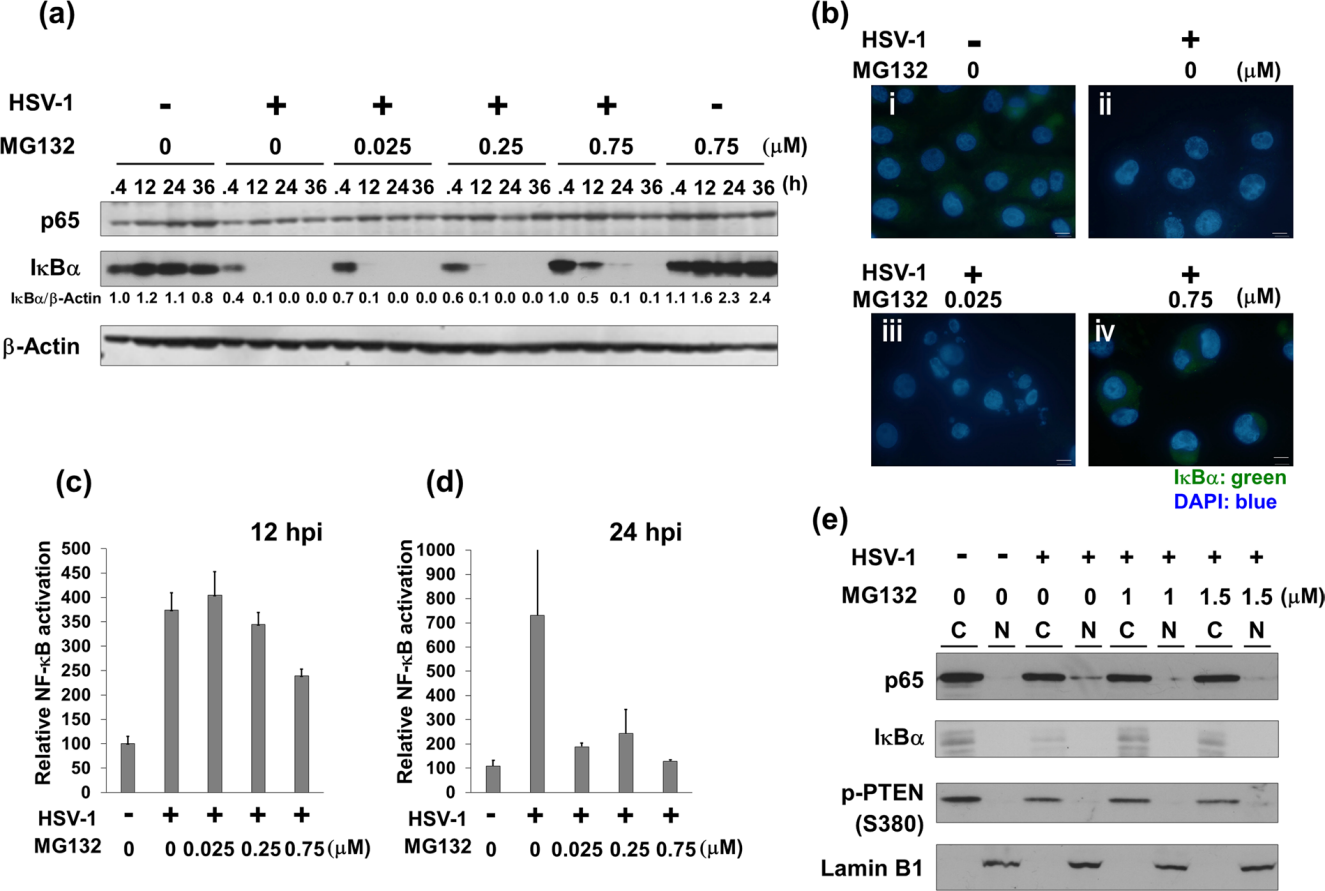
MG132 suppresses HSV-1-mediated NF-κB activation. (**a**) Effects of HSV-1 infection and MG132 treatment on NF-κB signaling. Vero cells were infected with HSV-1 at an MOI of 1 and cultured in media in the presence or absence MG132 for 0.4, 12, 24, or 36 hours. Whole-cell lysates were subjected to immunoblotting with an anti-IκBα or -NF-κB (p65) antibody. The values of IκBα/β-Actin are presented at the bottom of the image, and the values of 0.4 h nontreated and noninfected cells are presented as 1.0. (**b**) Immunofluorescence analysis using anti-IκBα antibody. Vero cells were mock infected (i) or HSV-1 infected at an MOI of 1 (ii, iii, iv) for 30 min and were treated with DMSO (i, ii), 0.025 μM MG132 (iii) or 0.75 μM MG132 (iv) for 12 hours. Cells were fixed with 4% PFA for 20 min and permeabilized with 0.1% Triton X-100 in PBS for 10 min, followed by incubation with anti-IκBα (green) antibody and DAPI to stain the nuclei (blue). Cells were observed by a fluorescence microscope (IX71) with a 60x oil-immersion objective. Scale bars represent 20 μm. The single channel gray images of IκBα are shown in Supplementary Fig. S3. (**c**,**d**) MG132 inhibits HSV-induced NF-κB transcriptional activity. HeLa cells were transfected with the NF-κB (p65) reporter plasmid and cultured for 18 hours. Transfected cells were infected with HSV-1 at an MOI of 1 for 30 min and treated with various concentrations of MG132 for 12 hours (**c**) or 24 hours (**d**), followed by cell lysis for luciferase detection. The NF-κB activity of HSV uninfected and MG132-untreated cells was defined as 100 relative activity. Standard deviations were determined by three independent experiments and are indicated by the error bars. (**e**) Subcellular localization of p65 and IκBα in HSV-1 infected cells. Vero cells were infected with HSV-1 at an MOI of 1 and then cultured with 1.0 or 1.5 μM MG132 for 7 hours. Harvested cells were lysed, and cytoplasmic fraction (C) and nuclear fraction (N) were prepared. The p-PTEN and lamin B1 were used as the cytoplasmic marker and the nuclear marker, respectively. Original images of blotting data (**a** and **e**) are shown in Supplementary Fig. S3.

HSV-1 infection has been reported to affect cell signaling pathways^8–24^. To reveal the mechanism by which MG132 suppresses HSV-1 replication, we analyzed the effects of HSV-1 infection in the presence and absence of MG132 on these pathways. Vero cells were infected with HSV-1 for 30 min, and then cultured with MG132 for 0.4–36 hours, followed by Western blotting. HSV-infection markedly decreased the expression levels of IκBα, which is a negative regulator of NF-κB (p65), in MG132-untreated cells from 12 to 36 hours postinfection compared with control (mock infected and MG132 untreated) cells, however, MG132 partially reversed HSV-mediated IκBα downregulation (Fig. 4a). The levels of p65 were unchanged in both infected and uninfected cells. We next confirmed the levels and subcellular localization of IκBα in HSV-infected Vero cells in the presence and absence of MG132 (Fig. 4b). IF analysis showed that abundant IκBα proteins (green) were localized in the cytoplasm of untreated normal cells (i), whereas HSV-infected cells at 12 hpi showed lower levels of IκBα in the cytoplasm (ii). Expression levels of IκBα proteins in the HSV infected cells treated 0.025 μM MG132 at 12 hpi (iii) was lower than that in the HSV infection cells treated 0.75 μM MG132 at 12 hpi (iv). However, 0.75 μM MG132 treatment (iv) showed an increase in cytosolic IκBα in HSV-infected cells compared with untreated cells at 12 hpi (ii), which was in agreement with Western blot data (Fig. 4a). It should be noted that Supplementary Fig. S3 shows single channel gray scale images of IκBα. To avoid misinterpreting the IκBα signal, please refer to the supplementary data. In addition, we performed a reporter assay using a NF-κB reporter plasmid to confirm HSV-induced NF-κB activation and inhibition by MG132. HeLa cells were transfected with an NF-κB-specific luciferase reporter plasmid and then were infected with HSV-1, followed by incubation with or without MG132 for 12 (Fig. 4c) and 24 hours (Fig. 4d). The NF-κB activity in cells 12 and 24 hours after infection displayed 4- and 7-fold increases, respectively, compared to mock-infected cells. However, HSV-induced NF-κB transcriptional activation was inhibited by MG132 treatments. Next, we analyzed the effect of MG132 on nuclear localization of p65 in HSV-1 infected cells. HSV-1 infection induced the destabilization of cytoplasmic IκBα and increased the nuclear localization of p65 (Fig. 4e), indicating the activation of NF-κB signaling by HSV-1 infection. Upon MG132 treatment, HSV-1-induced IκBα destabilization and the p65 nuclear localization was suppressed. These results indicate that HSV-1 infection downregulates IκBα, resulting in the activation of NF-κB signaling, whereas MG132 counters this effect.

### MG132 has no effect on HSV-mediated activation of the caspase pathway

**Figure 5:**
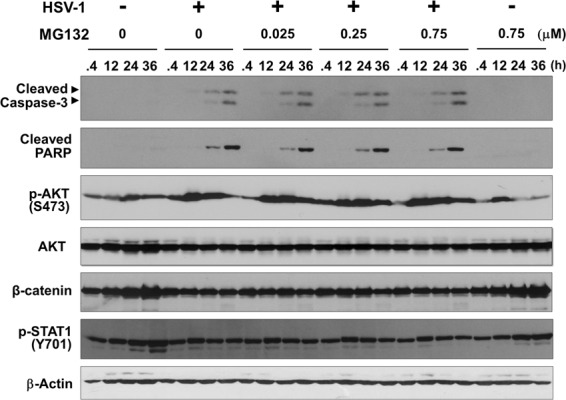
MG132 has no effect on HSV-mediated activation of the caspase pathway. Influence of HSV-1 infection and MG132 treatment on caspase, AKT, Wnt/β-catenin and STAT1 signaling was evaluated. Vero cells were mock or HSV-1 infected at an MOI of 1 for 30 min, and cultured with (or without) MG132 for 0.4, 12, 24, or 36 hours, followed by lysis in SDS-PAGE sample buffer and immunoblotting with antibodies against cleaved caspase-3, cleaved PARP, Ser473-phospho-AKT, AKT, β-catenin or Tyr701-phospho-STAT1. Two arrowheads indicate 19 kDa and 17 kDa of cleaved caspase-3, which are active forms, respectively. Original images of blotting data are shown in Fig. S4.

To elucidate the relationship between HSV-1 infection and apoptosis, the cleavage of caspase-3 and PARP, a substrate of caspase-3, was monitored by immunoblotting of lysates prepared from HSV-1 or mock infected Vero cells cultured with or without MG132 (Fig. 5). Although marked activation of caspase-3 and cleaved PARP were detected in HSV-1 infected cells, MG132 treatment did not affect HSV-induced cleavage of caspase-3 and PARP. We next investigated the effects of HSV-1 and MG132 on AKT, Wnt/β-catenin and STAT1 signaling pathways. When cells were infected with HSV-1, levels of phospho-AKT tended to increase while those of β-catenin tended to decrease compared with uninfected cells. However, the addition of MG132 did not influence these HSV-induced effects. Furthermore, no significant change in the level of phospho-STAT1 was detected in HSV-1 and mock infected cells regardless if MG132 was present or not.

### HSV-1-mediated suppression of ERK signaling is reversed by MG132-treatment

**Figure 6:**
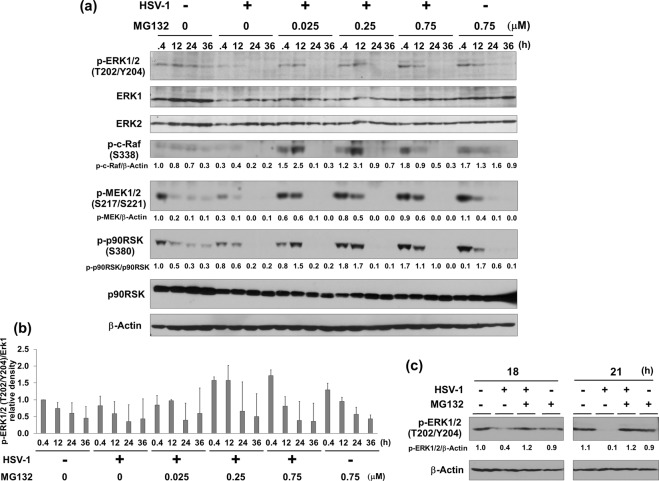
MG132 enhanced HSV-induced Raf-MEK-ERK-RSK cascade. (**a**,**b**) Effects of HSV-1 infection and MG132 treatment on ERK signaling. Vero cells infected with HSV-1 at an MOI of 1 or mock infected were cultured in media in the presence or absence MG132 for 0.4, 12, 24, or 36 hours. Whole-cell lysates were subjected to immunoblotting analysis with an anti-Thr202/Tyr204-phospho-ERK1/2, -ERK1, -ERK2, -Ser338-phospho-c-Raf, -Ser217/Ser221-phospho-MEK1/2, and –Ser380-phospho-p90RSK antibodies. The anti- Thr202/Tyr204 phospho-ERK1/2 antibody recognizes either Thr202- or Tyr204-phosphorylated ERK1 and also either Thr185- or Tyr187-phosphorylated ERK2. The values of p-c-Raf/β-actin, p-MEK/β-Actin and p-p90RSK/p90RSK are presented at the bottom of the image, and the values of 0.4 h nontreated and noninfected cells are presented as 1.0. (**b**) Densitometric analysis of the p-ERK1/2 in blotting data of (**a**). The quantitative analysis was performed using three separate membranes. The value of 0.4 h-nontreated and noninfected control was defined as 1.0. (**c**) Effects of HSV-1 infection and MG132 treatment on ERK signaling in human HepG2 cells. HepG2 cells infected with HSV-1 at an MOI of 1 and cultured with 0.75 μM MG132 for 18 and 21 hours. Cell lysates were subjected to immunoblotting using anti-Thr202/Tyr204 phospho-ERK1/2 antibody. The values of p-ERK1/2/β-Actin are presented at the bottom of the image, and the values of 18 h nontreated and noninfected cells are presented as 1. (**a**,**c**) Original images of blotting data are shown in Supplementary Fig. S5.

**Figure 7:**
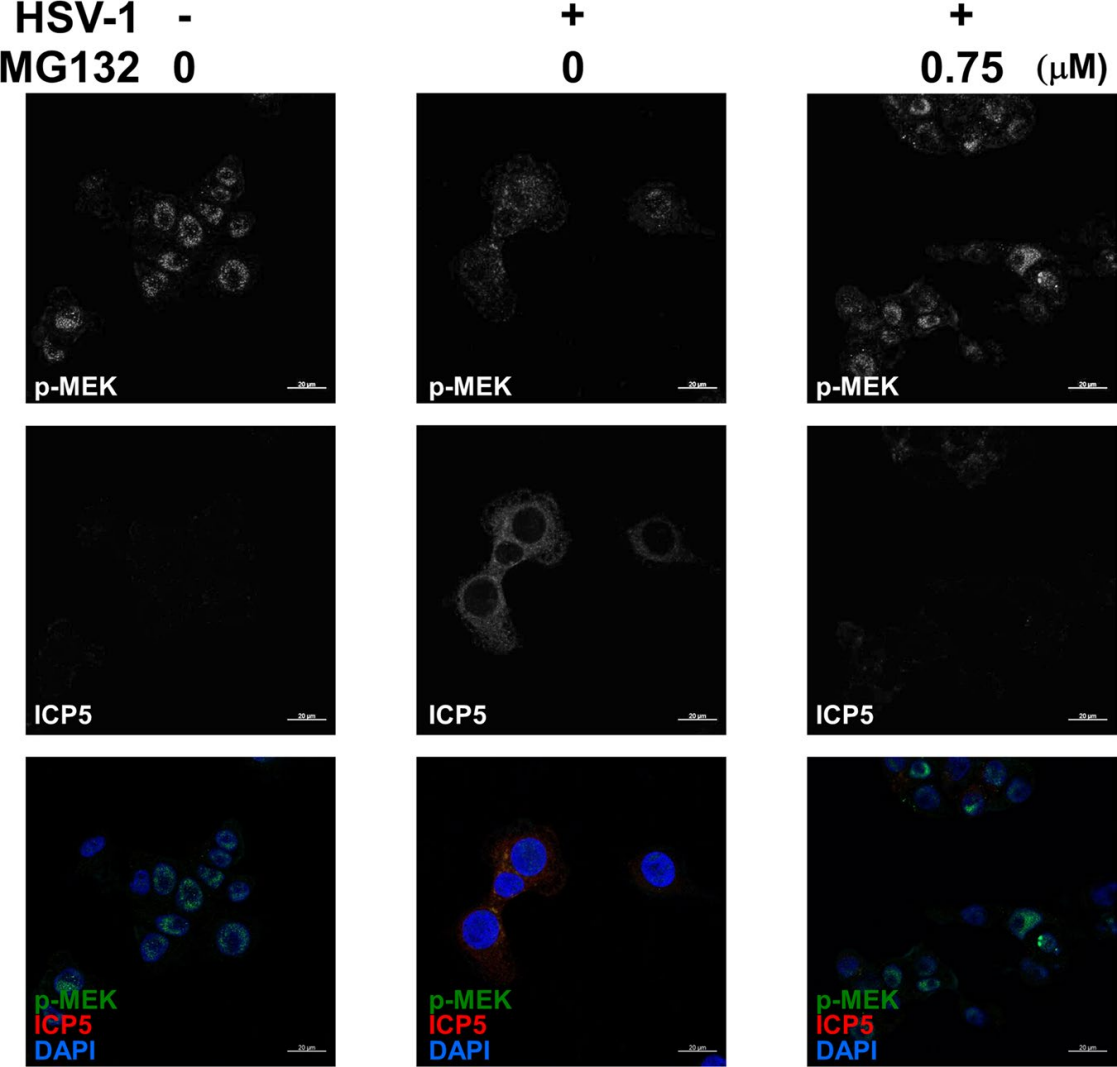
HSV-1 decreased phosphorylated MEK1/2, and MG132 increased cytoplasmic phosphorylated MEK1/2 in virus infected cells. Subcellular localization of the phospho-MEK1/2 in HSV-infected and MG132-treated cells. Vero cells were infected with HSV-1 at an MOI of 1 and treated with (or without) 0.75 μM MG132 for 10 hours. Vero cells were stained with anti-Ser217/Ser221-phospho-MEK1/2 (green), anti-ICP5 (red) and DAPI (blue) and observed by laser scanning confocal microscopy with a 40x water-immersion objective. Scale bars represent 20 μm. The upper and middle images show gray-scale images (single channel) of phospho-MEK1/2 and ICP5, respectively, and the lower images show triple staining of phospho-MEK1/2, ICP5 and DAPI.

We further investigated whether HSV-1 infection affects ERK signaling in the presence of MG132. When Vero cells were infected with HSV-1 for 30 min, phosphorylation levels of ERK1/2 were markedly decreased compared with mock-infected cells (Fig. 6a,b), however, MG132 treatment overcame HSV-induced ERK1/2 suppression at 0.4 and 12 hours postinfection. In addition to Vero cells, HSV-1 infection in human HepG2 cells also displayed same inhibitory effect on ERK1/2. Moreover, MG132 overcame HSV-induced ERK1/2 suppression not only in Vero but also HepG2 cells (Fig. 6c). HSV-1 infection and MG132 treatment did not affect the overall levels of ERK1 (p44 MAPK) and ERK2 (p42 MAPK). To obtain further evidence of HSV-mediated ERK inhibition and MG132-mediated ERK activation, we also checked ERK1/2 upstream regulators (c-Raf and MEK1/2)^35^ and the ERK substrate (p90RSK)^35^. Compared to control cells, HSV-1 infection decreased the phosphorylation of c-Raf, MEK1/2, and p90RSK (Fig. 6a). Interestingly, MG132 increased the phosphorylation of c-Raf, MEK1/2 and p90RSK in cells at 0.4 and 12 hours postinfection. These data indicate that HSV-1 infection inhibits ERK1/2 signaling through the suppression of upstream molecules c-Raf. Moreover, MG132-treatment was able to overcome HSV-1-induced ERK suppression. To validate MG132-mediated activation of the c-Raf-MEK-ERK pathway in HSV-1 infected cells, changes in localization and levels of phosphorylated MEK1/2 in Vero cells by MG132 treatment were observed by IF analysis (Fig. 7). As a result, faint levels of phosphorylated MEK1/2 in HSV-1 infected (ICP5-positive) and vehicle treated Vero cells were detected, while cytoplasmic phosphorylated MEK1/2 in mock infected and vehicle (DMSO) treated cells and in MG132 treated HSV-1 infected cells were detected.

### HSV-1 infection suppresses ERK signaling through proteasome-mediated degradation of Ras-GRF2

**Figure 8:**
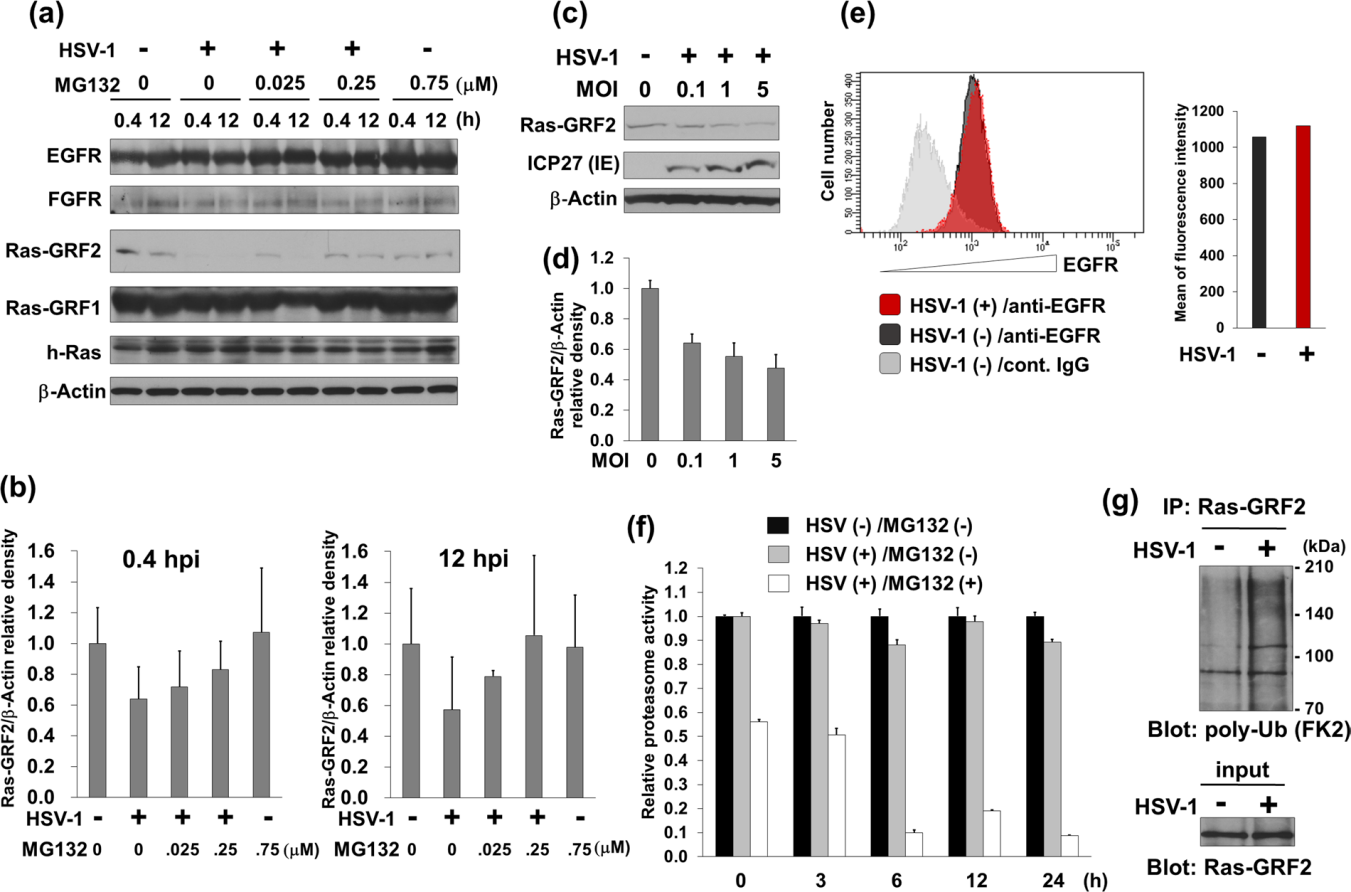
HSV-1 infection induced polyubiquitination of Ras-GRF2 and the proteasome-mediated destabilization of Ras-GRF2. (**a**) HSV-1 infection induces the proteasomal degradation of Ras-GRF2. Vero cells were mock-infected or infected with HSV-1 at an MOI of 1 and treated with MG132 for 0.4 or 12 hours. Cell lysates were analyzed by Western blotting. (**b**) Densitometric analysis of the Ras-GRF2 expression in blotting data of (**a**). The left graph and the right graph represents the band intensities of Ras-GRF2/β-Actin at 0.4 hpi and 12 hpi, respectively. The value of Ras-GRF2 in the virus- and drug-untreated control was defined as 1.0. Standard deviations were determined by three independent experiments and Western blotting. (**c**) Effects of HSV-1 MOI on the downregulation of Ras-GRF2. Vero cells infected with HSV-1 at various MOIs were cultured for 12 hours. (**d**) Densitometric analysis of Ras-GRF2 in (**c**). The value of Ras-GRF2 in the mock-infected control was defined as 1.0. Standard deviations were determined by three independent experiments and blotting. (**e**) The expression of EGFR in HSV-1 infected cells. The expression of EGFR was measured by flow cytometry. Gray histogram indicates the isotype control. Red and black histogram show EGFR expressing cells in HSV-1 infected cells and uninfected cells, respectively (left panel). The mean of fluorescence intensity of uninfected cells and HSV-1 infected cells are shown in a black and red bar graph, respectively (right panel). (**f**) Influence of HSV-1 infection and MG132 treatment on the proteasome activity. Vero cells were mock-infected or infected with HSV-1 at an MOI of 1 and were cultured with (or without) 0.75 μM MG132 for 0–24 hours. To measure the chymotrypsin-like activities of the proteasome, cell lysates were analyzed by a fluorometric assay. The proteasome activity of uninfected (HSV-) MG132-untreated (MG132-) cells, HSV-infected (HSV+) MG132-untreated (MG132-) cells, and HSV-infected (HSV+) MG132-treated (MG132+) cells are shown in a black, gray, and white bar graph, respectively. The activity of uninfected and MG132-untreated cells at 0 hpi  was defined as 1.0. (**g**) HSV-1 induced the polyubiquitination of Ras-GRF2. HSV-1-infected and -uninfected  cells were treated with 0.025 μM MG132 for 12 hours and, cell extracts were incubated with anti-Ras-GRF2 antibody-immobilized protein-A/G beads. To detect polyubiquitination of Ras-GRF2, immunoprecipitates were subjected to blotting with anti-polyubiquitin antibody (FK2 antibody). (**a**,**c**,**g**) Original images of blotting data are shown in Supplementary Fig. S6.

**Figure 9:**
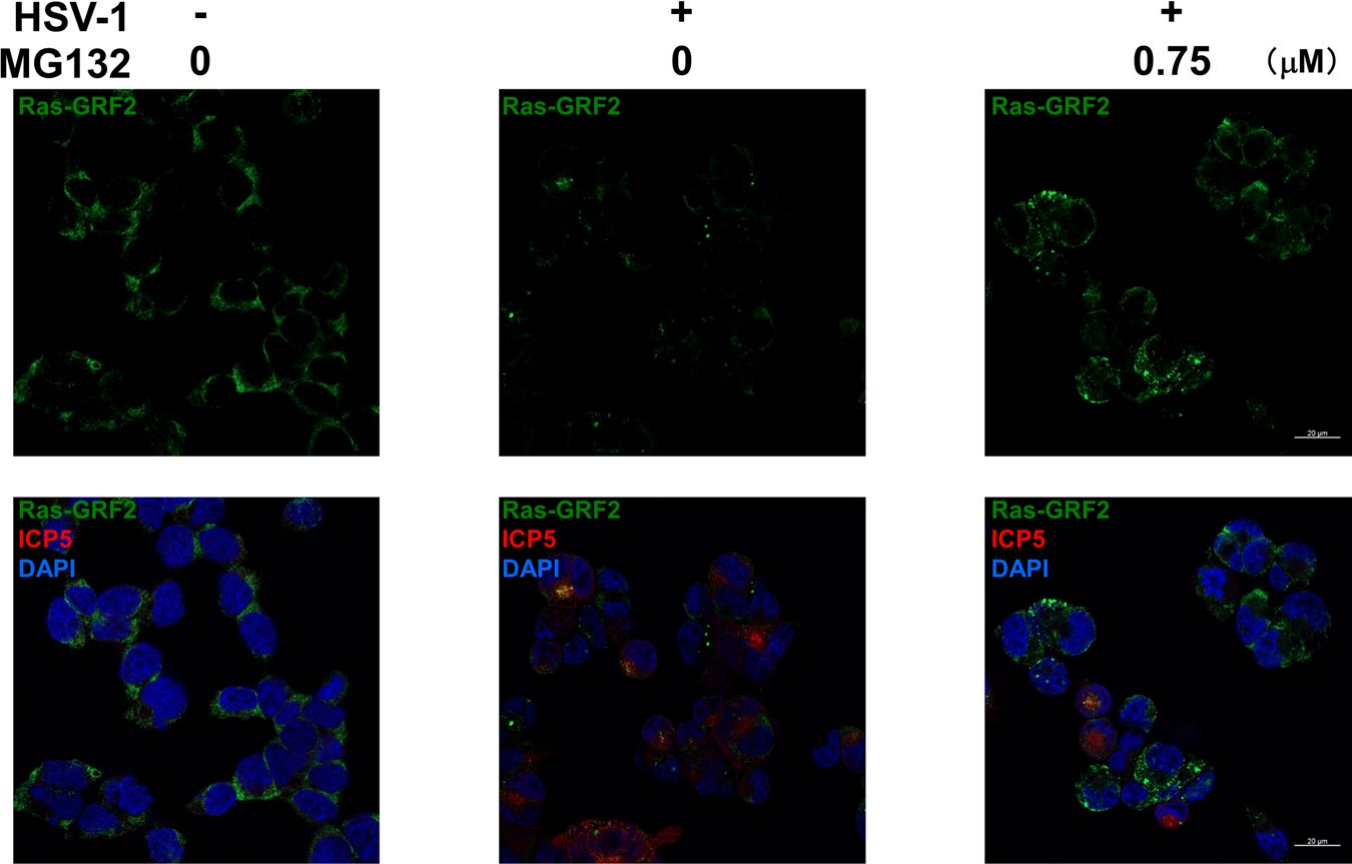
MG132 increased cytoplasmic Ras-GRF2 in HSV-infected cells. HepG2 cells were infected with HSV-1 at an MOI of 1 and treated with 0.75 μM MG132 for 12 hours. Cells were stained with anti-Ras-GRF2 (green), anti-ICP5 (red) and DAPI (blue) and observed by laser scanning confocal microscopy with 40x water-immersion objective. Upper images show the Ras-GRF2 staining, and lower images show triple staining of Ras-GRF2, ICP5 and DAPI. Scale bars represent 20 μm.

We found that HSV-1 infection suppressed the c-Raf-MEK1/2-ERK1/2-p90RSK pathway in host cells, while proteasome inhibitor MG132 activated this pathway. On the other hand, the proteasome has been known to degrade EGF-receptor (EGFR)^36,37^ and Ras-guanine nucleotide-releasing factor 2 (Ras-GRF2)^38^, which is an activator of Ras. To obtain insight into the mechanism by which HSV-mediated suppression of ERK signaling is reversed by MG132-treatment, we investigated the effects of HSV-1 and MG132 on the expression of upstream regulators of c-Raf. Vero cells infected with HSV-1 were treated with MG132, and EGFR, h-Ras, Ras-GRF1 and Ras-GRF2 were evaluated by Western blotting. HSV-1 infection and MG132 treatment had no influence on the expression of Ras-GRF1, h-Ras and EGFR. However, HSV-1 infection remarkably induced downregulation of Ras-GRF2 at 0.4–12 hours postinfection, and MG132 treatment partially inhibited this downregulation (Fig. 8a,b), indicating a proteasome-mediated degradation of Ras-GRF2. In addition, HSV-1 induced Ras-GRF2 downregulation in an MOI-dependent manner (Fig. 8c,d). We also performed flow cytometry analysis to monitor the expression level of EGFR in HSV-1 infected Vero cells, indicating that the amount of EGFR was not decreased in HSV-1 infected cells compared with untreated cells (Fig. 8e). The mechanism of Ras-GRF2 downregulation could occur in one of two ways: (i) the activation of the proteasome, which can degrade polyubiquitinated proteins; and (ii) an increase in the polyubiquitination of Ras-GRF2. We attempted to determine which event is enhanced by HSV-1 infection, leading to the downregulation of Ras-GRF2. First, the chymotrypsin-like activity of the proteasome was measured with a fluorogenic peptide substrate. However, there was no difference in the proteasome activity between HSV-1 infected cells and mock infected cells (Fig. 8f). On the other hand, we could confirm that MG132 strongly inhibited the proteasome activity of treated cells in a time dependent manner. Next, to examine whether HSV-1 infection increased the polyubiquitination of Ras-GRF2, polyubiquitinated Ras-GRF2 in HSV-1 infected and mock infected cells was monitored by co-immunoprecipitation. As a result, the polyubiquitination of Ras-GRF2 was remarkably increased in HSV-1 infected cells compared with uninfected cells (Fig. 8g). These data indicate that HSV-1 infection induces the polyubiquitination of Ras-GRF2 and subsequently degradation of Ras-GRF2 by the proteasome, leading to suppression of ERK signaling. In addition, we validated the expression and localization of Ras-GRF2 in HSV-infected HepG2 cells with (or without) MG132 (Fig. 9). IF analysis showed that Ras-GRF2 proteins (green) were localized in the cytoplasm of untreated and uninfected cells, whereas HSV-infected cells showed lower levels of Ras-GRF2 proteins. MG132 treatment showed an increase in Ras-GRF2 in HSV-infected cells compared with untreated cells, which was in agreement with Western blot data (Fig. 8a).

### Inhibition of ERK signaling enhances HSV-1 multiplication while inhibition of NF-κB has no effect on HSV-1 replication

**Figure 10:**
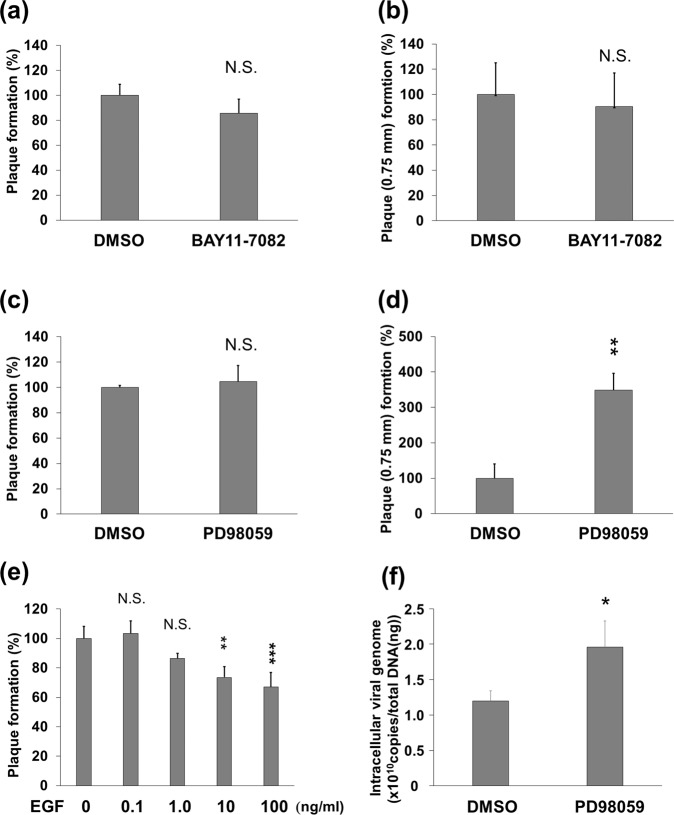
The suppression of ERK but not NF-kB signaling by small chemical inhibitors contributes to HSV-1 replication. (**a**–**e**) Effect of NF-κB inhibitor (BAY11-7082), ERK inhibitor (PD98059) and EGF on HSV-1 multiplication. Vero cells were infected with HSV-1 at 100 PFU for 30 min, and cultured with DMSO (**a**–**d**) or sterile water (**e**), 1 μM BAY11-7082 (**a**,**b**), 10 μM PD98059 (**c**,**d**) or 0.1–100 ng/ml EGF (**e**) for 48 hours, followed by plaque analysis. (**a**,**c**,**e**) and (**b**,**d**) display the total number of plaques and the number of the plaques with a diameter of over 0.75 mm, respectively. The number of plaques of untreated cells was defined as 100%. The number of plaques of DMSO or sterile water treated cells was defined as 100%. (**f**) Effect of PD98059 on HSV-1 proliferation in human HepG2 cells. HepG2 cells were infected with HSV-1 at an MOI of 1 for 30 min and subsequently cultured with DMSO or 10 μM PD98059 for 20 hours. Infected cells were harvested, and genomic DNA containing viral DNA was isolated and measured by real-time PCR. (**a**–**f**) Standard deviations were determined by analyzing the data from three experiments. ^*^P < 0.05, ^**^P < 0.01 and ^***^P < 0.001 indicate the statistical significance compared with vehicle (DMSO) treated cells, and N.S. indicates not significant.

HSV-1 infection activated NF-κB but suppressed ERK1/2 signaling (Figs. 4 and 6), whereas MG132-treatment suppressed NF-κB but activated the ERK signaling pathway. To determine whether the anti-HSV activity of MG132 can be attributed to its modulation of signaling pathways, we examined the effects of selective signaling inhibitors or activator on HSV-1 replication. Cells infected with HSV-1 were cultured with NF-κB inhibitor (BAY11-7082), ERK inhibitor (PD98059) or epidermal growth factor (EGF) for 2 days, followed by quantification of plaques, which result from cytopathic effects during HSV-1 replication. BAY11-7082, which inhibits IκB phosphorylation by IKK complex, is an irreversible and specific inhibitor of NF-κB signaling, while PD98059 inhibits MEK1/2, resulting in a specific inhibition of ERK signaling. EGF was used as an activator of ERK1/2 signaling. We measured the total number of plaques (Fig. 10a,c,e) and the number of the plaques with a diameter greater than 0.75 mm (Fig. 10b,d). As a result, the inhibition of the ERK signaling pathway did not affect total plaque numbers (Fig. 10c), whereas the inhibition of NF-κB signaling decreased the total number of plaques by about 10% compared to vehicle treatment (Fig. 10a). Interestingly, plaque sizes increased significantly upon inhibition of ERK with PD98059, whereas NF-kB inhibition had no effect on plaque sizes (Fig. 10b). PD98059 also significantly increased the number of plaques (over 0.75 mm) by approximately 3.5-fold, compared with DMSO control (Fig. 10d). Moreover, EGF, used as an activator of the Ras-ERK signaling, decreased the total number of plaques in a concentration dependent manner (Fig. 10e). In addition to plaque quantification, we evaluated the effect of PD98059 on intracellular viral DNA replication in HepG2 cells. Cells infected with HSV-1 were cultured with ERK inhibitor (PD98059) for 20 hours, followed by quantification of intracellular HSV-1 DNA. Intracellular viral replication increased approximately 2-fold in PD98059-treated cells (Fig. 10f). These results indicate that the suppression of ERK signaling facilitates viral replication, but suppression of NF-κB signaling has no effect. The activation of ERK signaling pathways has potent anti-HSV-1 activity and results in a reduction of virus yields.

## Discussion

Here, we demonstrate that the proteasome inhibitor (MG132 and bortezomib) inhibited HSV-1 replication, and MG132 also inhibited lytic gene expression, suggesting that MG132 impairs an early step in the replication cycle before lytic gene expression. Bortezomib (Velcade) is also a proteasome inhibitor and an FDA approved drug for multiple myeloma and mantle cell lymphoma. MG132 is a reversible and cell membrane permeable inhibitor of the proteasome which degrades polyubiquitinated proteins and plays a role in numerous cellular events such as the regulation of signaling pathways, apoptosis, cell cycling, and antigen presentation through degradation of substrate proteins^25,36^.

The proteolytic activities of the proteasome during the HSV life cycle have been known to exert multiple effects and is nessesary for HSV and the host cell. Proteasome inhibition by MG132 or Bortezomib has been reported to influence the infection establishment and viral replication as well as the anti-viral response. The inhibitors were reported to suppress proteasome activity, which was nessesary for an early step of HSV infection such as virus entry. Delboy *et al*. showed that MG132 blocked an early step in HSV entry that occurred after capsid penetration into the cytosol but prior to capsid arrival at the nucleus^33^. They also found a relationship between E3 ubiquitin ligase activity of the HSV immediate early protein ICP0 and HSV-infection, and further proposed that proteasome mediated degradation of unknown viral or host proteins is regulated by ICP0 to permit efficient delivery of incoming HSV capsids to the nucleus^33,34^. Everett *et al*. found that HSV-1-encoded IE protein, Vmw110, induced disruption of ND10 (also known as PML nuclear bodies) in a proteasome dependent manner during early HSV infection^39^. They also found that MG132 inhibited Vmw110-stimulated viral gene expression and HSV-1 reactivation^40^. Proteasome inhibition by bortezomib was reported to affect early HSV infection by disrupting two proteasome-dependent stages that occur within the initial hours of infection, resulting in exertion of anti-HSV activity^41^. Bortezomib inhibited the transport of incoming viral nucleocapsids to the nucleus and the HSV-induced disruption of nuclear domain 10 (ND10) structures^41^. In addition to ND10 disruption and transport of viral capsid, proteasome function was required to localize ICP0 to the cytoplasm from nucleus, and this translocation was suppressed by MG132^42^. The cytoplasmic ICP0 inhibited IRF3 activation and IRF3-dependent ISG induction at later stages of infection. Moreover, proteolytic activities of the proteasome during the HSV-1 infection have been known to be involved in various viral events including inactivation of antiviral response such as degradation of the tegument protein VP11/12 (UL46) in an ICP0-dependent manner^43^, degradation of the transcriptional repressor TRIM27 in an ICP0-dependent manner^44^, degradation of the interferon-inducible protein X (IFIX) recognized as an antiviral factor^45^ and degradation of TANK binding kinase 1 (TBK1) in a Us11 (dsRNA-dependent protein kinase/PKR viral inhibitory protein)-dependent mannre^46^. These HSV-1-induced proteasomal degradation phenomena were blocked by MG132 treatment. In addition to HSV-1, Pastenkos *et al*. showed that ammonium chloride and monensin, which block the acidification of endosomes, and MG132 inhibited Bovine herpesvirus 1 (BoHV-1) entry in a concentration-dependent manner^47^. Yakoub *et al*. showed that autophagy induction by MG132-treatment and starvation suppressed HSV-1 infection^48^. MG132 treatment and starvation are known to induce accumulation of aggregates consisting of denatured proteins and subsequently formation of autophagosomes. They proposed that HSV-1 fails to infect autophagy activated cells^48^.

Proteasome functions are necessary for multiple viral events during the HSV life cycle. Importantly, MG132 has been reported to inhibit these proteasome-dependent events. Although we found that MG132 overcame HSV-1-mediated suppression of the ERK signaling, MG132 affects not only ERK signaling but also various proteasome-dependent events as mentioned above. It is possible that the anti-viral effect by MG132 is owing to more complex modes of inhibition.

**Figure 11:**
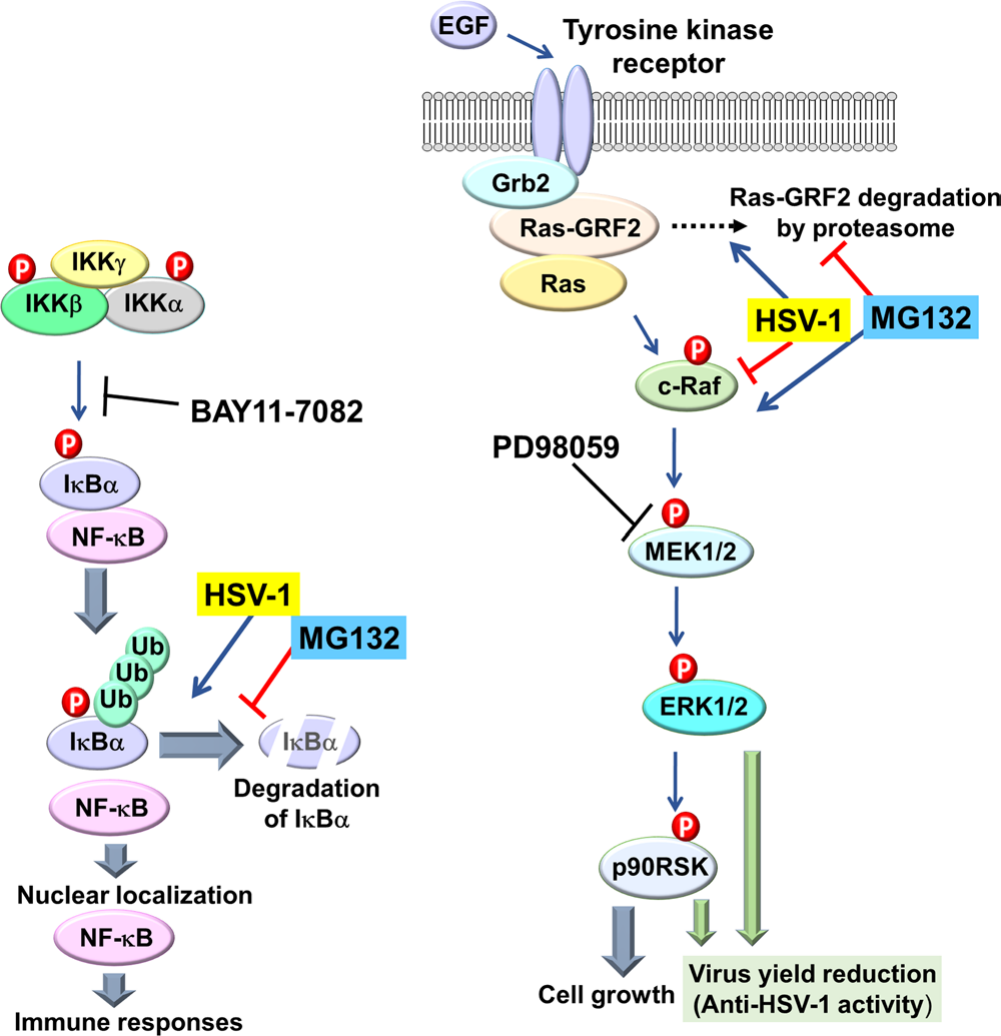
Model of alteration of NF-κB and ERK signaling pathways during HSV-1 infection and MG132-treatment. When an HSV-1 virion infects the host cell, it activates NF-κB signaling through IκBα destabilization, but suppresses c-Raf-MEK1/2-ERK1/2 signaling by proteasome-mediated downregulation of Ras-GRF2. However, MG132-treatment during HSV-infection suppresses HSV-induced NF-κB activation, whereas MG132 overcomes HSV-1 suppression of ERK signaling. The inhibition of ERK by PD98059 facilitates viral replication, whereas NF-κB suppression by BAY11-7082 has no effect. In sum, MG132 activates ERK signaling and thereby reduces viral replication, demonstrating anti-HSV-1 activity. HSV-1-mediated NF-κB activation might be involved in cellular functions such as innate immune responses.

Here, we found that HSV-1 infection activated NF-κB signaling, but suppressed ERK signaling in host cells. There are above-mentioned reports showing the relationship between MG132 and suppression of virus entry. In contrast, we disclosed that MG132 treatment during HSV infection suppressed NF-κB activation, whereas MG132 overcame HSV-1-mediated suppression of ERK signaling. Furthermore, HSV-1 infection suppresses ERK signaling, which is due to HSV-1-induced proteasomal degradation of Ras-GRF2 that is an upstream activator of ERK signaling. ERK suppression facilitated viral replication, whereas NF-κB suppression did not affect viral replication. Activation of ERK signaling by MG132 strongly contributed to a reduction in virus yields (i.e., anti-HSV-1 activity). We propose a model by which MG132 exerts an anti-HSV-1 effect through MG132-dependent activation of ERK signaling (Fig. 11). HSV infection-induced ERK dephosphorylation is consistent with previous reports by Ian Mohr and colleagues^19,21^, which showed ERK dephosphorylation was detectable 9 hpi (MOI of 5) and depended on viral gene expression. However, we observed ERK dephosphorylation after 0.4 hpi (MOI of 1). Ian Mohr’s group evaluated ERK dephosphorylation in growth arrested normal human dermal fibroblast cells by serum-deprivation, whereas our study used serum, which may be the cause of the discrepancy in the time of ERK dephosphorylation after HSV-1 infection. In addition, Colao *et al*. showed that HSV-1 infection in HEp-2 cells increased cytoplasmic phospho-ERK1/2 at 0.25–0.5 hpi to 2- to 1.4-fold compared to those of mock infection, whereas cytoplasmic phospho-ERK1/2 decreased to 10% after 24 hpi compared^49^. Interestingly, HSV-1 infection increased nuclear phospho-ERK1/2 at 3–9 hpi to 3- to 4-fold. They proposed that virus-induced ERK phosphorylation contributed to controlling the cell cycle progression from G1 to S phase and viral replication^49^. These difference may be owing to differences in the genetic background of tested cells or amount of growth factors in culture medium. Although, we did not validate cytoplasmic fractionated phospho-ERK1/2 in this study, previous published findings^49^ that phospho-ERK1/2 markedly reduced after 24 hpi are partially in agreement with our observations.

We also found that HSV-infection induced activation of caspase signaling at 24–36 hpi (Fig. 5). There are reports indicating that HSV-infection induced apoptosis of infected host cells. Whereas HSV was reported to inhibit apoptosis at an early and middle stage of infection^50,51^. The HSV-1 regulatory proteins (ICP4 and ICP27) have an ability to block apoptosis by promoting the production of anti-apoptotic viral gene products, and LATs, gD, gJ, and Us3 directly (or indirectly) inhibit apoptosis related molecules^50,51^. We observed many fusion cells at 36 hpi and some dying cells detached from the culture plate. As cells after 24 hpi were producing virions (Fig. 3i), cells around 36 hpi were in the late stage of lytic replication. Thus, detection caspase-3 and PARP cleavage at 24–36 hpi is understandable and reasonable.

For many viruses, incoming viral tegument proteins during initial virus infection and lytic gene products manipulate transcriptional signaling pathways in the host cell. In particular, the ERK signaling pathway has been utilized by many viruses to establish infection, stimulate their replication, regulate cell proliferation, and suppress apoptosis^15,16^. In general, the binding of EGF to EGFR activates the tyrosine kinase activity of the cytoplasmic domain of the receptor (Fig. 11). Grb2 interacts with the phosphotyrosine residues of the activated EGFR and also interacts with the guanine-nucleotide exchange factors (GEFs) such as Ras-GRF or SOS^35^. Ras-GRF2 is a GEF that activates Ras- and Rac-dependent ERK signaling and contains a PEST motif for degradation and a Cdc25 domain, which possesses GEF activity and interacts with Ras^52,53^. When Grb2 and GEF form a complex and interact with phosphorylated EGFR, the GEF becomes activated and then promotes the conversion of the GDP-bound Ras into the GTP-bound form (active Ras)^35^. The activated Ras mediates the phosphorylation of Ser 338 of c-Raf, which activates MEK1/2 through the Ser217/Ser221 phosphorylation of MEK1/2. Activated MEK1/2 mediates Thr202/Tyr204 phosphorylation and activation of ERK1/2, which in turn phosphorylates abundant substrates including p90RSK^35^. During ERK dysregulation by HSV-1, two inconsistent findings have been reported: (i) HSV-infection suppresses ERK signaling for viral replication in an infected cell^19–21^, and (ii) HSV-infection activates ERK signaling for viral replication^22–24^. We observed not only the decrease in phosphorylation of c-Raf, MEK1/2, ERK1/2, and p90RSK but also a decrease in Ras-GRF2 expression by HSV-1 infection, which means that HSV-1 infection inactivates ERK signaling through the downregulation of Ras-GRF2 in host cells, lending support to previously published results^19–21^. Furthermore, ERK suppression by PD98059 increased the size of plaques and the number of plaques over 0.75 mm, indicating that the suppression of ERK signaling facilitates viral replication. MG132 treatment after HSV-infection significantly increased Ras-GRF2 levels and the phosphorylation of c-Raf, MEK1/2, ERK1/2 and p90RSK, indicating that MG132 inhibits HSV-induced proteasomal degradation of Ras-GRF2 and furthermore reverses HSV-1 mediated suppression of ERK signaling. Thus, it seems reasonable to conclude that the suppression of ERK signaling through proteasome-mediated degradation of Ras-GRF2 is necessary for HSV-1 infection and/or replication, however, MG132 reverses HSV-1 mediated suppression of ERK signaling by inhibiting proteasomal degradation of Ras-GRF2 and exerts anti-HSV-1 activity.

On the other hand, Ras-GRF2 and EGFR have been known to be polyubiquitinated and degraded by the proteasome^37,38^. Ras-GRF2 has a PEST motif containing a destruction box for polyubiquitination of Ras-GRF2 and a Cdc25 domain for interaction with Ras^52,53^. The destruction box of Ras-GRF2 and the binding to Ras via a Cdc25 domain of Ras-GRF2 are necessary for polyubiquitination and proteasomal degradation of Ras-GRF2^38^. In the case of EGFR, c-Cbl (henceforth designated Cbl) functions as an E3 ubiquitin ligase and determines the fate of the receptor^37,54^. The polyubiquitinated EGFR is endocytosed in a clathrin-dependent manner and degraded by the lysosome^54^. In addition to the lysosome pathway, it has been known that the polyubiquitination of EGFR is mediated by c-Cbl^37^ or Hsp90-CHIP^55^, and polyubiquitinated EGFR is degraded by the proteasome. In fact, we demonstrated that HSV-1 infection induces polyubiquitination and degradation of Ras-GRF2, but not Ras and EGFR (Fig. 8a,e). Furthermore, the proteasome inhibitor MG132 inhibited HSV-1-induced degradation of Ras-GRF2. Hence, it is interesting to speculate that a HSV protein such as ICP0 could act as an E3 ubiquitin ligase for Ras-GRF2, leading to the proteasome degradation of Ras-GRF2. It is also possible that HSV-1 induces the activation of c-Cbl or Hsp90-CHIP for Ras-GRF2 polyubiquitination.

NF-κB signaling is triggered early after innate immune activation. This pathway is activated by the binding of HSV PAMPs with TLR2, 4 and 9 during infection^13,14^. HSV dependent TLR activation induces the phosphorylation of IKKβ and IKKα through TRAF6 or a TAK1–TAB2 complex, and activates the IKK complex leading to the phosphorylation of IκBα. Phosphorylated IκBα is polyubiquitinated and subsequently downregulated by the proteasome (Fig. 11). This degradation leads to NF-κB nuclear translocation and transcription of inflammatory cytokine genes^8–10^. In addition to the TLRs pathway, TLR-independent NF-κB activation by HSV-1 UL37 protein was disclosed^56^. UL37 protein leads to IκBα degradation and contains a TRAF6-binding domain that is required for interaction with TRAF6 and activation of NF-κB^56^. Furthermore, previous reports have shown HSV-mediated upregulation of NF-κB leads to successful virus replication and viral gene expression^57,58^. We confirmed the activation of NF-κB signaling after HSV-1 infection, and also demonstrated that MG132 suppressed HSV-1-induced NF-κB activation by inhibiting the proteasomal degradation of IκBα. Diao *et al*. showed that MG132 inhibits ICP0-induced activation of NF-κB^59^. In addition, other proteasome inhibitors (MG115) and resveratrol were also reported to inhibit viral protein expression and viral replication by suppressing NF-κB signaling^60,61^. Thus, we expected MG132 to similarly have anti-HSV activity via NF-κB suppression. However, the inhibition of NF-κB by BAY11-7082 did not affect viral replication, indicating that this pathway is dispsensable for the establishment of HSV-1 infection and HSV-1 replication. The Western blotting data showed that IκBα was completely degraded in HSV-infected cells in the presence of 0.025 and 0.25 µM MG132 at 24 hpi (Fig. 4a), whereas the NF-κB reporter assay showed that HSV-induced NF-κB transcriptional activities were not so high in 0.025 and 0.25 µM MG132-treated cells at 24 hpi (Fig. 4d). We speculate that unknown viral or cellular inhibitory factors, which might inhibit NF-kB-transcriptional activity, were produced after 12 or 24 hpi in infected cells. Those inhibitory factors could inhibit NF-kB signaling in an IκBα-independent manner or inhibit NF-kB transcription factor at the downstream of IκBα-mediated suppression.

In conclusion, we demonstrate that HSV-1 infection suppresses the Ras-Raf-MEK-ERK signaling pathway in host cells through the polyubiquitination and proteasomal degradation of Ras-GRF2. The proteasome inhibitor, MG132, inhibits HSV-1 replication by impairing HSV-1-mediated ERK suppression. Our study sheds new light on the mechanisms through which HSV-1 perturbs cellular signaling pathways, resulting in successful replication. Furthermore, our findings suggest that proteasome inhibitors may be applicable for HSV-1 treatment.

## Methods

### Reagents, cells and viruses

Tosyllysine chloromethyl ketone (TLCK), Tosylphenylalanyl chloromethyl ketone (TPCK), E64, and Z-Leu-Leu-Leu-CHO (MG132) were purchased from Peptide Institute (Osaka, Japan). MEK1/2 inhibitor (PD98059), NF-kB inhibitor (BAY11-7082), and bortezomib were obtained from Merck (NJ, USA), and dissolved in dimethyl sulfoxide (DMSO). Recombinant epidermal growth factor (EGF) was purchased from FUJIFILM Wako Pure Chemical Corporation (Osaka, Japan) and dissolved in sterile water. Vero, HepG2, H1299, ME180, MCF7 and HeLa cells were maintained in DMEM (high glucose) supplemented with 10% fetal calf serum  (FCS). The HF strain of herpes simplex virus type 1 (HSV-1) was used for infection experiments.

### Plaque reduction assay

The inhibitory activities of compounds against HSV-1 were determined by the plaque reduction assay. Vero cells (2 × 10^5^ cells/well), seeded in a 12-well tissue culture plate, were cultivated for one day, and the cell monolayers were once washed with phosphate buffered saline (PBS). Vero cells were inoculated with 50 plaque forming units (PFU) of HSV-1 in 0.1 ml of DMEM without FBS. After a 30 min inoculation, the inoculum was removed, and HSV-1 infected cells were maintained with DMEM containing 10% FCS, 1% methylcellulose, and serially diluted test compounds. After 48 hours incubation at 37 °C, the medium was removed. Then the cell sheets were stained with 1% crystal violet in 50% methanol, and the total number of plaques was counted or the number of plaques with a diameter over 0.75 mm was counted. HSV-1 replication results in cytopathic effects in the form of plaques. The number of plaques reflects how efficiently the virus establishes infection, and the size of plaques reflects how efficiently the virus spreads from cell to cell. To compare the efficiency of virus yield reduction, the total plaque number in compound-untreated cells was defined as 100%. Standard deviations were determined by analyzing the data from three experiments and are indicated by the error bars.

### Cell viability assay

Cells were seeded in 96-well plates at 5 × 10^3^ cells/well in 100 μl of medium with or without various concentrations of the inhibitor and then incubated at 37 °C for 36 hours. The number of viable cells was estimated using Cell Count Reagent SF (Nakalai tesque, Kyoto, Japan). The optical density at 450 nm of each sample was measured using a microplate spectrophotometer (Tecan M200; Tecan, Kanagawa, Japan) and expressed as a percentage of the value in untreated cells (defined as 100%).

### Western blotting and antibodies

Cells were seeded at 4 × 10^5^ cells per well in a 6-well plate and cultivated for 12 hours. Cells were infected with HSV-1 at a multiplicity of infection (MOI) of 1 for 30 min and subsequently cultured in the presence (or absence) of MG132 for 0.4, 12, 24 or 36 hours. Cells were lysed by 300 μl of 4x SDS-PAGE sample buffer (containing 4% 2-mercaptoethanol, 0.5 mM phenylmethylsulfonyl fluoride, 1 μg/ml pepstatin and 5 μg/ml aprotinin), boiled for 5 min, and sonicated for 30 sec with a probe type sonicator in order to shear the chromosomal DNA. The resulting lysate was subjected to SDS-PAGE on 8 or 12% polyacrylamide gel followed by Western blotting. The proteins were transferred onto a ClearTrans nitrocellulose membrane (FUJIFILM Wako) and the membrane was incubated with 3% non-fat dry milk in PBS containing 0.1% Tween-20 (PBS-T) for 1 h at room temperature. Then, the membrane was incubated with a primary antibody (750- or 1500-fold dilution) and subsequently with a secondary antibody (HRP-conjugated anti-mouse or anti-rabbit IgG antibody) (3000-fold dilution) in Can Get Signal Immunoreaction Enhancer Solution (Toyobo, Osaka, Japan). The membrane was incubated in ECL Western Blotting Detection Reagents (GE Healthcare Life Sciences, IL, USA), and visualized with X-ray film (FUJIFILM Wako). Primary antibodies used in these experiments were Ser380-phospho-PTEN, lamin B1, cleaved caspase-3, cleaved PARP, Ser473-phospho-AKT, AKT, Tyr701-phospho-STAT1, Ser338-phospho-c-Raf, Ser217/Ser221-phospho-MEK1/2, Thr202/Tyr204-phospho-ERK1/2, Ser380-phospho-p90RSK, FGFR, and EGFR (1:1500 dilution, Cell Signaling Technology, MA, USA), Ras-GRF2 (1:1500 dilution, Abcam, OR, USA), IκBα, p65, β-catenin, ERK1, and ERK2 (1:1500 dilution, BD Biosciences, NJ, USA), β-Actin, p90RSK, ICP5, ICP8, ICP27, UL42, Ras-GRF1, and h-Ras (1:750 dilution, Santa Cruz Biotechnology, CA, USA). The cross-reactivities of primary antibodies are shown in Supplementary Table S1. As cross-reactivities of several antibodies were unknown, we confirmed their cross-reactivity by Western blotting using human and monkey cell extracts (Fig. S1).

### Cell fractionation

Vero cells seeded at 2 × 10^6^ cells per well in a 10 cm dish plate were infected with HF strain at an MOI of 1 for 30 min and subsequently cultured in the presence (or absence) of MG132 for 7 hours. Cells were incubated with 400 μl of hypotonic buffer (10 mM HEPES at pH 7.9, 10 mM KCl, 0.1 mM EDTA, 0.1 mM EGTA, 1 mM dithiothreitol and 0.5 μM phenylmethylsulfonylfluoride) for 15 min on ice and lysed by addition of NP-40 (final concentration, 0.6%). After heavy agitation with a vortex mixer, cell lysates were centrifuged at 15,000 rpm for 30 sec at 4 °C. Harvested supernatants (400 μl) were mixed with 100 μl of 5x SDS-PAGE sample buffer and were used as cytoplasmic fraction. The nuclear pellets were rinsed two times with the hypotonic buffer containing 0.6% NP-40. Washed nuclear pellets were lysed by 50 μl 1x SDS-PAGE sample buffer and used as nuclear fraction.

### Measurement of extracellular and intracellular viral production

For quantification of extracellular virus production^62^, cells (2 × 10^5^ cells/well) seeded in 12-well dish were infected with HSV-1 at an MOI of 1 for 30 min and subsequently cultured with the drug for 24 hours, except for Vero cells. Vero cells were cultured with drug for 12 hours. Culture supernatants (220 μL) were harvested and treated with DNase I (NEB, MA, USA) to obtain enveloped and encapsidated viral genomes. Supernatant was heated at 95 °C for 7 min for inactivation of DNase I, and viral DNA was purified and extracted from 200 μL of heated supernatant using the QIAamp DNA blood mini kit (QIAGEN, CA, USA). To quantify viral DNA copies, SYBR green real-time PCR was performed using HSV-1 UL19 specific primers (UL19-forward 5′-AACAGCCTGTACGACGTC-3′ and UL19-reverse 5′-AACTTGGTACACACGCACGC-3′). To generate a standard curve for cycle threshold versus genomic copy number, the UL19-inserted pGEM-T Easy plasmid (Promega, WI, USA) was serially diluted to known concentrations in the range of 10^4^ to 10^10^ plasmid molecules/µl. For quantification of intracellular virus production^62^, cells (2 × 10^5^ cells/well) were infected with HSV-1 at an MOI of 1 for 30 min and subsequently cultured with the drug for 43 hours (Fig. 3f) or 20 hours (Fig. 10f). Total cellular DNA containing the HSV-1 genome was purified and extracted from HSV-1 infected cells using the QIAamp DNA blood mini kit (QIAGEN). Intracellular HSV-1 genome copies were determined by SYBR green real-time PCR using above-mentioned UL19 primers and normalized to the total DNA concentration.

### RT real-time PCR (RT-qPCR)

HepG2 cells (2 × 10^5^ cells/well) were infected with HF strain at an MOI of 1 for 30 min and subsequently cultured in the presence (or absence) of MG132 for 20 hours. mRNA was extracted from HSV-1 infected cells using RNAiso Plus (Takara, Osaka, Japan). cDNA was synthesized by Revetra Ace qPCR kit (Toyobo, Oaka, Japan) and subjected to SYBR green real-time PCR^62^. Relative mRNA expression levels were determined by GAPDH expression and ΔΔCt methods. The sequences of oligonucleotides used for RT-qPCR primers were as follows: Us12-forward 5′-AGATCGTAGTGTCCGCACCG-3′, Us12-reverse 5′-CTTAAAAGGCGTGCCGTCCG-3′, UL19-forward 5′-AACAGCCTGTACGACGTC-3′ and UL19-reverse 5′-AACTTGGTACACACGCACGC-3′) The primer set for GAPDH (forward 5′-CATCAAGAAGGTGGTGAAGCAG-3′ and reverse 5′-TGTCGCTGTTGAAGTCAGAGG-3′) was used as an internal control for normalization. For quantification, the expression level of each gene was normalized to that of the GAPDH gene.

### Attachment assay

Vero cells growing in 12 well culture plates were prechilled at 4 °C for 5 min and were infected with 200 PFU/well of HSV-1 in the presence of the drug at 4 °C for 30 min. The medium was aspirated, and cells washed with PBS two times to remove unadsorbed virus. Culture medium containing 1% methylcellulose without drug were overlaid on cell monolayer, and cells were cultured for 2 days. Cells were stained with 1% crystal violet, and the total number of plaques was counted.

### Penetration assay

Vero cells growing in 12 well culture plate were prechilled at 4 °C for 5 min followed by infection with 100 PFU/well of HSV-1 for 30 min at 4 °C. After removing the virus and washing cells, cells were incubated with the drug for 30 min at 37 °C to allow penetration. Cells were washed with citrate buffer (135 mM NaCl, 10 mM KCl, 40 mM sodium citrate, pH 3.0) to remove cell surface virus, and the cell monolayer was washed with PBS two times. Culture medium containing 1% methylcellulose without drug was overlaid on a cell monolayer, and cells were cultured for 2 days. Cells were stained with 1% crystal violet, and the total number of plaques was counted.

### One-step growth curve

HepG2 cells were infected with HF strain (1 PFU/cell) for 30 min and subsequently washed with culture media two times. Cells were cultured in DMEM containing 10% FBS supplemented with MG132 and ACV. The culture media was harvested at 0, 6, 12, 24 and 48 hours postinfection and stored at −80 °C. The amount of produced virus in the culture media was measured by a plaque reduction assay using Vero cells.

### Immunofluorescence (IF) analysis

Prior to immunofluorescence (IF) analysis^62^, Vero or HepG2 cells were grown on coverslips and infected with (or without) HSV-1 at an MOI of 0.1 or 1 for 30 min. Cells were fixed with 4% paraformaldehyde (PFA) in PBS for 20 min at room temperature and permeabilized with 0.1% Triton X-100 in PBS for 10 min. After blocking with 10% FCS in PBS-T for 0.5 hours, the cells were incubated with a primary antibody in PBS and subsequently with Alexa Fluor 488 or 594-conjugated donkey anti-mouse (or rabbit IgG) in PBS (BD Biosciences). To stain the nuclei, 4′-6-Diamidino-2-phenylindole (DAPI) was stained using Fluoro-KEEPER Antifade Reagent, Non-Hardening Type with DAPI (Nacalai). All of the primary antibodies were employed at a 1:100 dilution, and the secondary antibodies were applied at a 1:500 dilution. Immunofluorescence images were obtained by a fluorescence microscope (IX71; Olympus, Tokyo, Japan) or a confocal microscope (LSM800 with Airyscan; Carl Zeiss, Oberkochen, Germany) with 40×/NA 1.2 water-immersion objective.

### Luciferase reporter assay

HeLa cells (1 × 10^5^) were transfected with 2 μg of a NF-κB-luciferase reporter plasmid (pGL4.32; Promega), together with 1 μg of a pSV-β-Gal plasmid (Promega) by Chen and Okayama calcium-phosphate method^63^. pSV-β-Gal was used as an internal control for transfection efficiency. After 18 hours, transfected cells were infected with HSV-1 at an MOI of 1 for 30 min and cultured in media containing MG132 for 12 or 24 hours. Cells were resuspended in 0.1 ml of lysis buffer for luciferase and β-Gal analysis^54^. Luciferase activity was measured with a GloMax 20/20 luminometer (Promega). The luciferase activity divided by β-galactosidase activity in virus- and drug-untreated cells was defined as 100%.

### Flow cytometry

Vero cells were removed from culture dishes with 0.05% trypsin in 5.5 mM EDTA, and washed with PBS containing 3% FBS. Removed cells were fixed in 4% PFA for 10 minutes and permeabilized using 0.1% Triton X-100 for 30 minutes. After washing with PBS containing 3% FBS, cells were incubated with a primary antibody in PBS containing 3% FBS for 1 hour at 4 °C and subsequently incubated with Alexa Fluor 647 anti-mouse secondary antibody for 1 hour. Cells washed with PBS containing 3% FBS were subjected to analysis by a LSRFortessa flow cyctometer (BD Biosciences) with FACSDiva software (BD Biosciences).

### Co-immunoprecipitation

Immunoprecipitation assays to detect polyubiquitinated proteins were performed as previously published^64^. Vero cells (1 × 10^7^) were lysed in 2 ml RIPA buffer (PBS containing 0.1% SDS, 0.5% deoxycholic acid, 1% Nonidet P-40, 0.1 mM EDTA, 1 mM N-ethylmaleimide, 0.5 mM phenylmethylsulfonyl fluoride, and 5 μg/ml aprotinin). Cell lysates (1.2 ml) were incubated with 20 μl protein A/G-Sepharose beads, which were immobilized with 1.5 μg of anti-Ras-GRF2 antibody for 1 hour. Beads were washed with ice-cold RIPA buffer three times. Then, resulting beads were resuspended in 30 μl sample buffer, 15 μl of which was subjected to SDS-PAGE followed by blotting with FK2 (anti-polyubiquitin) antibody that we established before^65^.

### Measurement of proteasome activity

Vero cells (1 × 10^6^) were lysed in 0.2 ml of buffer (50 mM Tris-HCl (pH 7.6), 1 mM MgCl_2_, 0.2 mM ATP, 1 mM DTT, 0.1 mM EDTA, 0.2% NP-40 and 1% glycerol) and homogenized with 27 G needles and 1 ml disposable syringes. The protein concentrations of cell lysates were measured by BCA method using a Protein Assay Bicinchoninate Kit (Nacalai Tesque, Kyoto, Japan). The proteasome activity in cell lysates was assessed with a fluorogenic peptide, Suc-Leu-Leu-Val-Tyr-4-methylcoumaryl-7-amide (MCA) (Peptide Institute, Osaka, Japan). The cell lysates (10 μl) were incubated in 90 μl of reaction buffer containing 50 mM Tris-HCl (pH 7.8), 10 mM MgCl_2_, 2 mM ATP, 1 mM DTT, 0.1 mM Suc-Leu-Leu-Val-Tyr-MCA. The fluorescence intensity owing to MCA (excitation, 380 nm: emission, 460 nm) was determined using a microplate spectrofluorometer Infinite M200 (Tecan).

### Densitometry and statistical analysis

Quantitative densitometric measurement of immunoblotting was performed using ImageJ software (NIH, MD, USA). Standard deviations were determined by analyzing the data from at least three independent experiments and are indicated by the error bars. The statistical significance was analyzed by one-way ANOVA followed by Dunnett’s test for multiple comparisons (Figs. 1d,e, 2d,e, 3a–e and 10e) or the two-tailed Student’s *t*-test (Figs. 3f–h and 10a–d,f). Statistically significant data were analyzed with GraphPad Prism 7 (GraphPad Software, CA, USA). ^*^P < 0.05, ^**^P < 0.01 and ^***^P < 0.001 indicate the statistical significance compared with vehicle (DMSO) treated cells. N.S. indicates not statistically significant.

## Supplementary information

Supplementary Information.
