# Supplementary material for: MG132 exerts anti-viral activity against HSV-1 by overcoming virus-mediated suppression of the ERK signaling pathway
Source: Sci Rep. 2020 Apr 21;10:6671. doi: 10.1038/s41598-020-63438-1 (PMC7174428; doi:10.1038/s41598-020-63438-1)

Supplementary information

MG132 exerts anti-viral activity against HSV-1 by overcoming virus-mediated suppression of the ERK signaling pathway

Hanako Ishimaru, Kohei Hosokawa, Atsuko Sugimoto, Riho Tanaka, Tadashi Watanabe and Masahiro Fujimuro*

Department of Cell Biology, Kyoto Pharmaceutical University,
Misasagi-Shichono-cho 1, Yamashina-ku, Kyoto 607-8412, Japan.

*: Address correspondence to Masahiro Fujimuro, PhD.
Department of Cell Biology, Kyoto Pharmaceutical University
Misasagi-Shichono-cho 1, Yamashina-ku, Kyoto 607-8412, Japan;
Tel: +81-75-595-4717
E-mail: fuji2@mb.kyoto-phu.ac.jp

Supplementary Table S1: The cross-reactivities of the primary antibodies

Antibody	Isotype	Species Cross-Reactivity[a]	Company	catalog #
ICP27	Mouse IgG	HSV-1, HSV-2	Santa Cruz	sc-69807
ICP5	Mouse IgG	HSV-1, HSV-2	Santa Cruz	sc-56989
β-Actin	Mouse IgG	broad species	Santa Cruz	sc-69879
UL42	Mouse IgG	HSV-1	Santa Cruz	sc-53331
p65	Mouse IgG	R, Dg, R, Ra	BD Biosciences	610869
IκBα[b]	Mouse IgG	H, Mk	BD Biosciences	610691
p-PTEN(S380)	Rabbit IgG	H, M, R	Cell Signaling	9551
Lamin B1	Rabbit IgG	H, M, R, Mk, Pg	Cell Signaling	12586
Cleaved Caspase-3	Rabbit IgG	H, M, R, Mk, B, Dg, Pg	Cell Signaling	9661
Cleaved PARP[b]	Rabbit IgG	H, Mk	Cell Signaling	9541
p-AKT(S473)	Rabbit IgG	H, M, R, Mk	Cell Signaling	13038
AKT	Rabbit IgG	H, M, R, Mk, Dm,Pg	Cell Signaling	4691
β-Catenin	Mouse IgG	M, R, Dg, C	BD Biosciences	610154
p-STAT1(Y701)	Rabbit IgG	H, M, R, B, Dg	Cell Signaling	9171
p-ERK1/2(T202/Y204)	Rabbit IgG	H, M, R, Mk, Mi, Pg, Sc, Hm, B, Dm, Z, Dg, C	Cell Signaling	4370
ERK1	Mouse IgG1	H, M, C, Dg, F	BD Biosciences	610031
ERK2	Mouse IgG	H, Mk, M, X, Sc, Z	BD Biosciences	51-9001959
p-MEK1/2(S217/S221)	Rabbit IgG	H, M, R, Mk,C	Cell Signaling	9154
p-c-Raf(S338)	Rabbit IgG	H, M, R, Mk	Cell Signaling	9427
p-p90RSK(S380)	Rabbit IgG	H, M, R, Mk,Mi,C, X, Z, B, Dg, Pg, Hr	Cell Signaling	11989
EGFR	Rabbit IgG	H, M, Mk	Cell Signaling	4267
FGFR	Rabbit IgG	H, M, R, Mk	Cell Signaling	9740
Ras-GRF2[b]	Rabbit IgG	H, Mk	abcam	ab 121577
Ras-GRF1	Mouse IgG	H, M, R	Santa Cruz	sc-377234
h-Ras	Mouse IgG	H, M, R	Santa Cruz	sc-35
FK2 (anti-polyubiquitin)[c]	Mouse IgG	H, Mk, M, X, Sc, Z		

[a] H, human; M, mouse; R, rat; Mk, monkey; Dm, Drosophila melanogaster; X, Xenopus laevis; Pg, pig; Sc, S. cerevisiae; Hm, hamster; B, bovine; Z, zebrafish; Dg, dog; C, chicken; Mi, mink; Hr, Horse; F, Frog; Ra, rabbit.

[b] We confirmed the cross-reactivity by Western blotting (Data were shown in Supplementary Fig. S1).

[c] Our laboratory established it before[65].

Supplementary Fig. S1

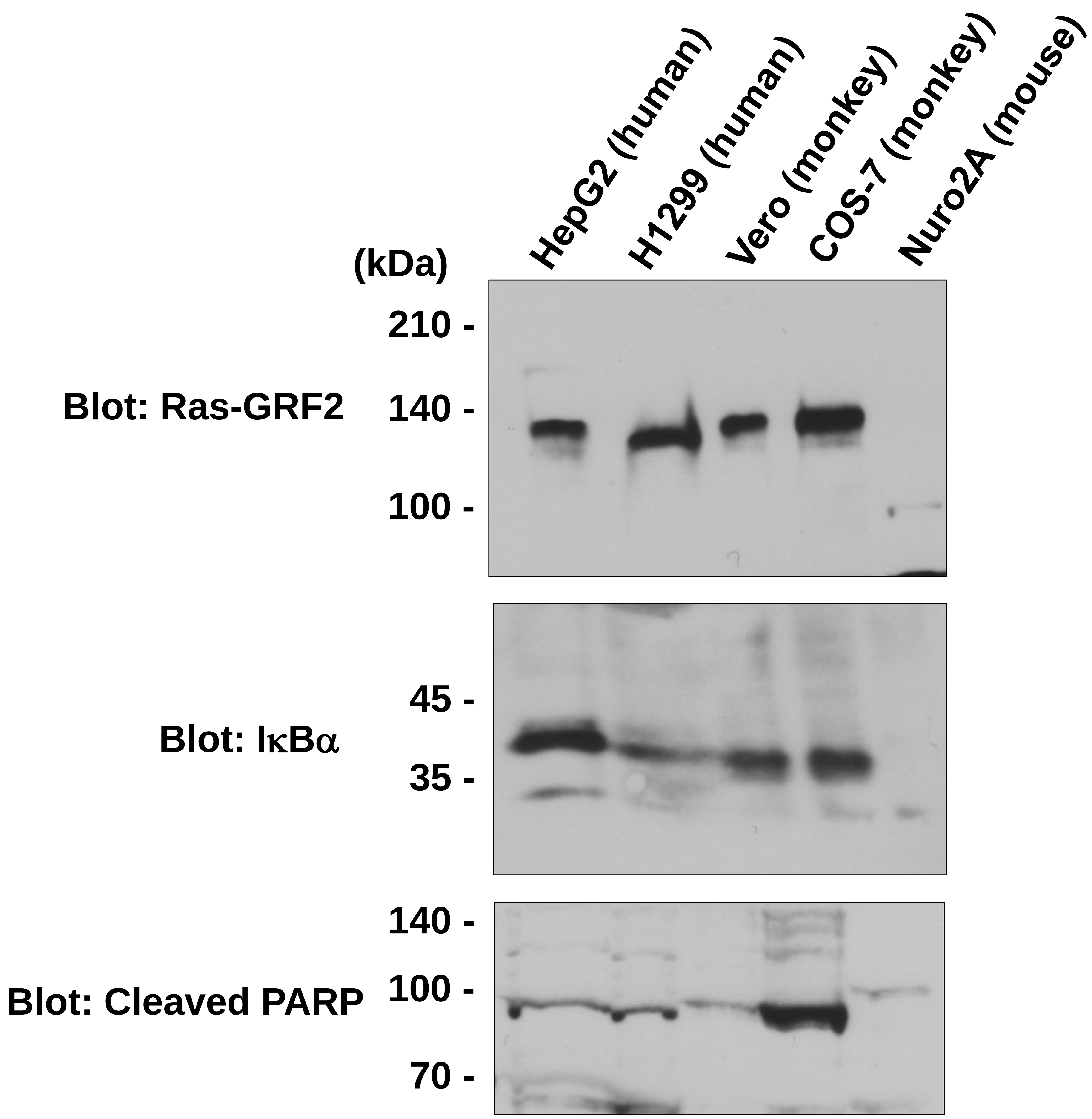

The validation of the cross-reactivities of Ras-GRF2, IκBα and cleaved PARP antibodies by Western blotting. HepG2 (human), H1299 (human), Vero (monkey), COS-7 (monkey) and Nuro2A (mouse) cells were cultured in DMEM with 10% fetal calf serum. Harvested cells ($1x10^6$ cells) were lysed by 300 μl of 4x SDS-PAGE sample buffer containing 4% 2-mercaptoethanol, boiled for 5 min, and sonicated for 30 sec. Whole-cell lysates were subjected to SDS-PAGE on 8% polyacrylamide gel followed by Western blotting.

Supplementary Fig. S2 (original data of Figure 2a, 2b and 2c)

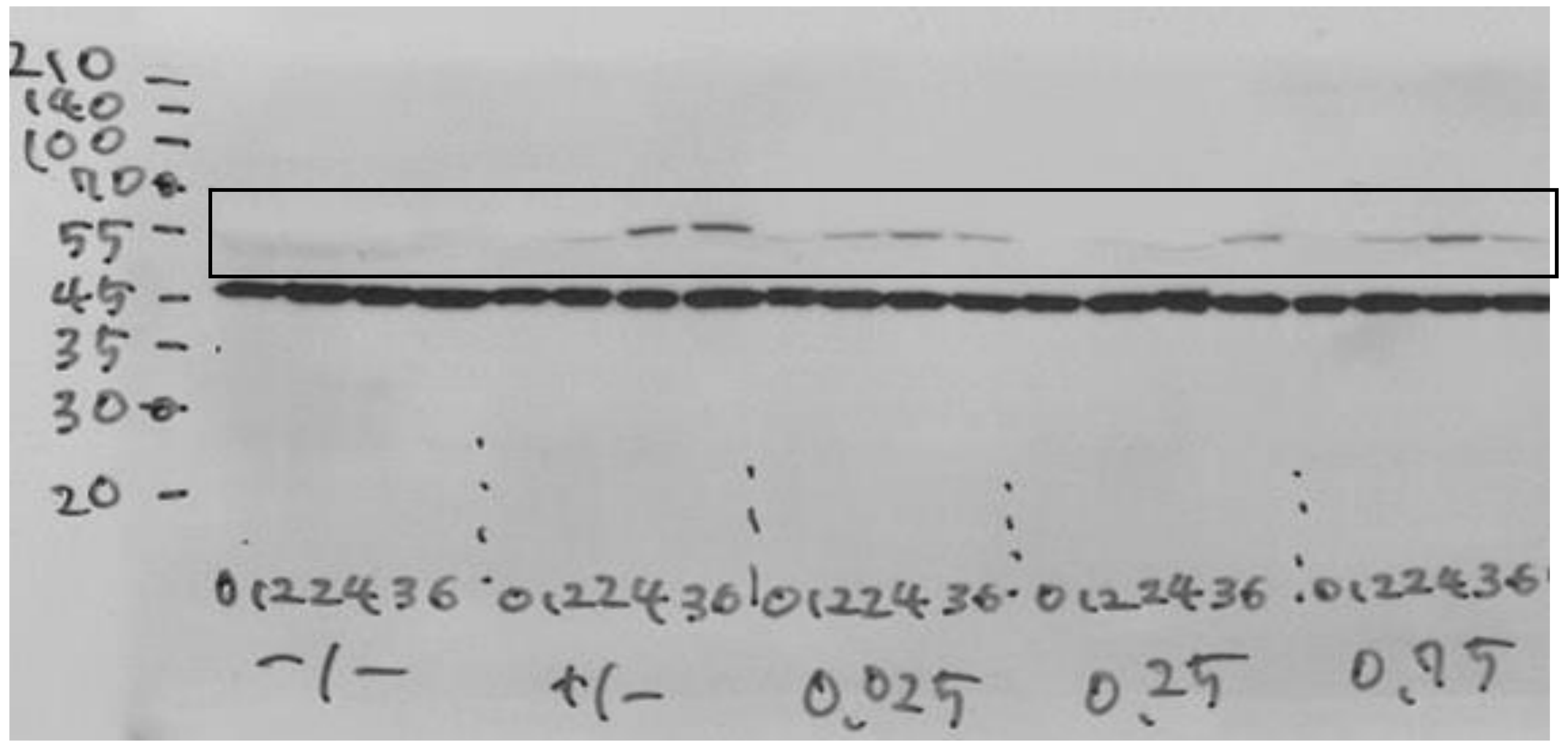

Blot: ICP27 and following Blot: β-actin

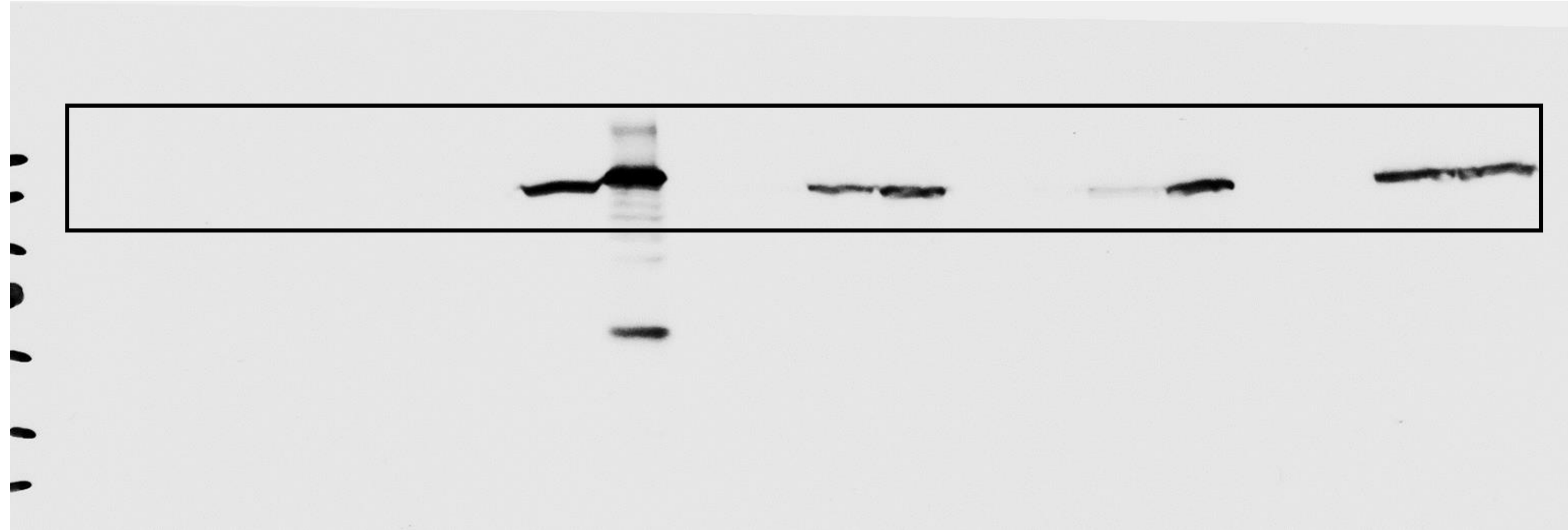

Blot: ICP5

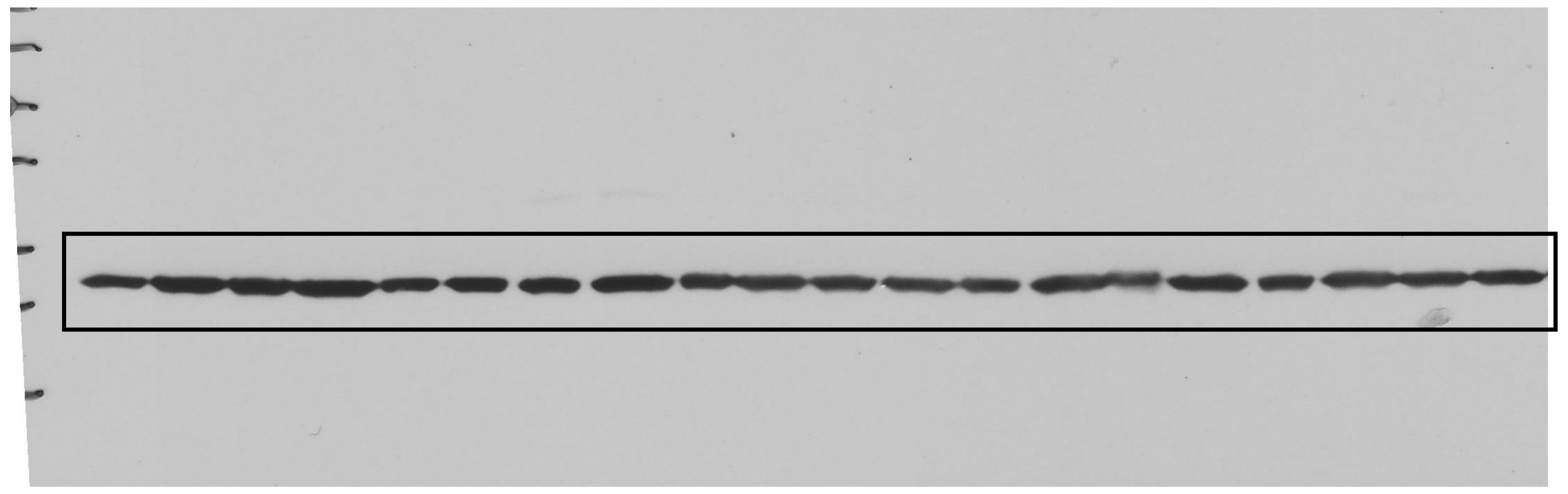

Blot: β-Actin

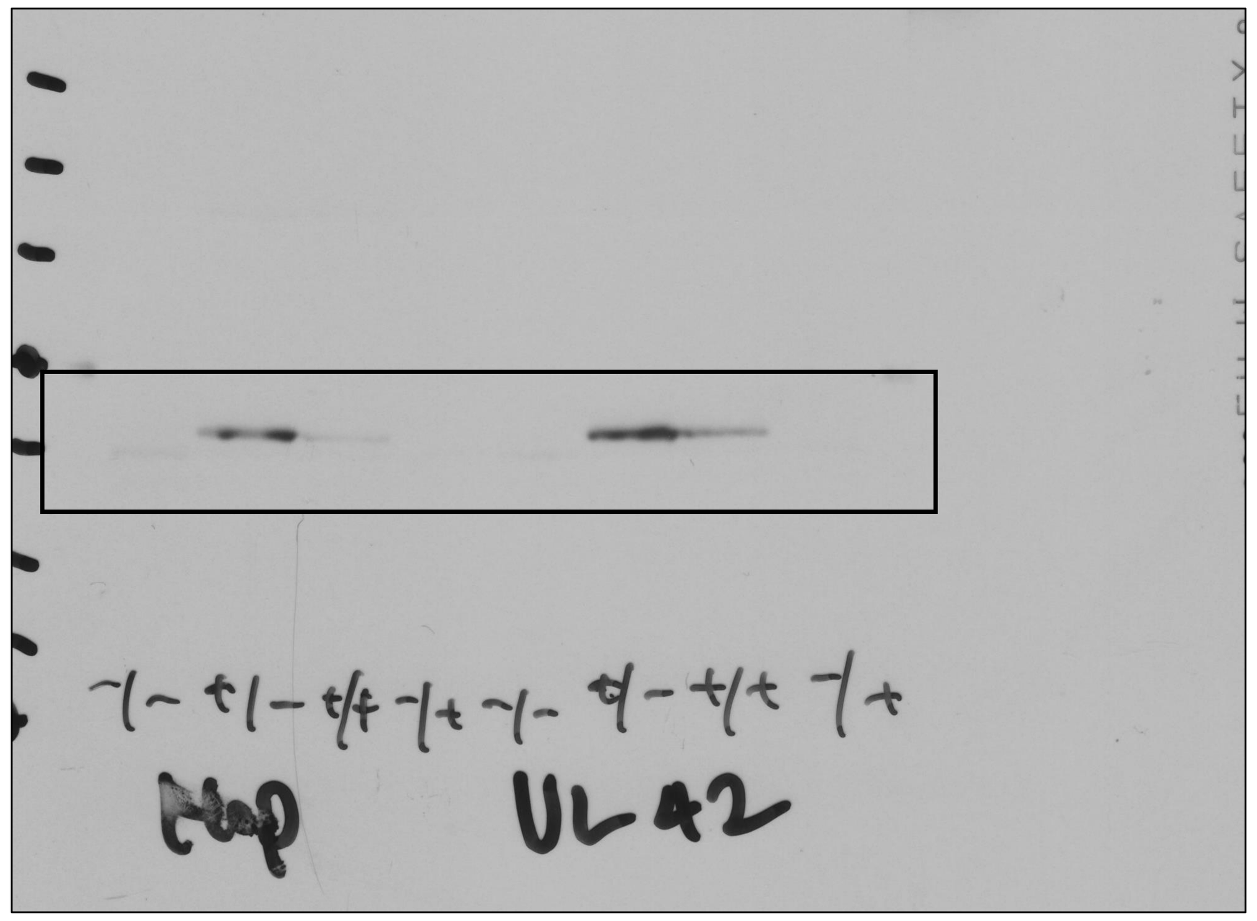

Blot: UL42

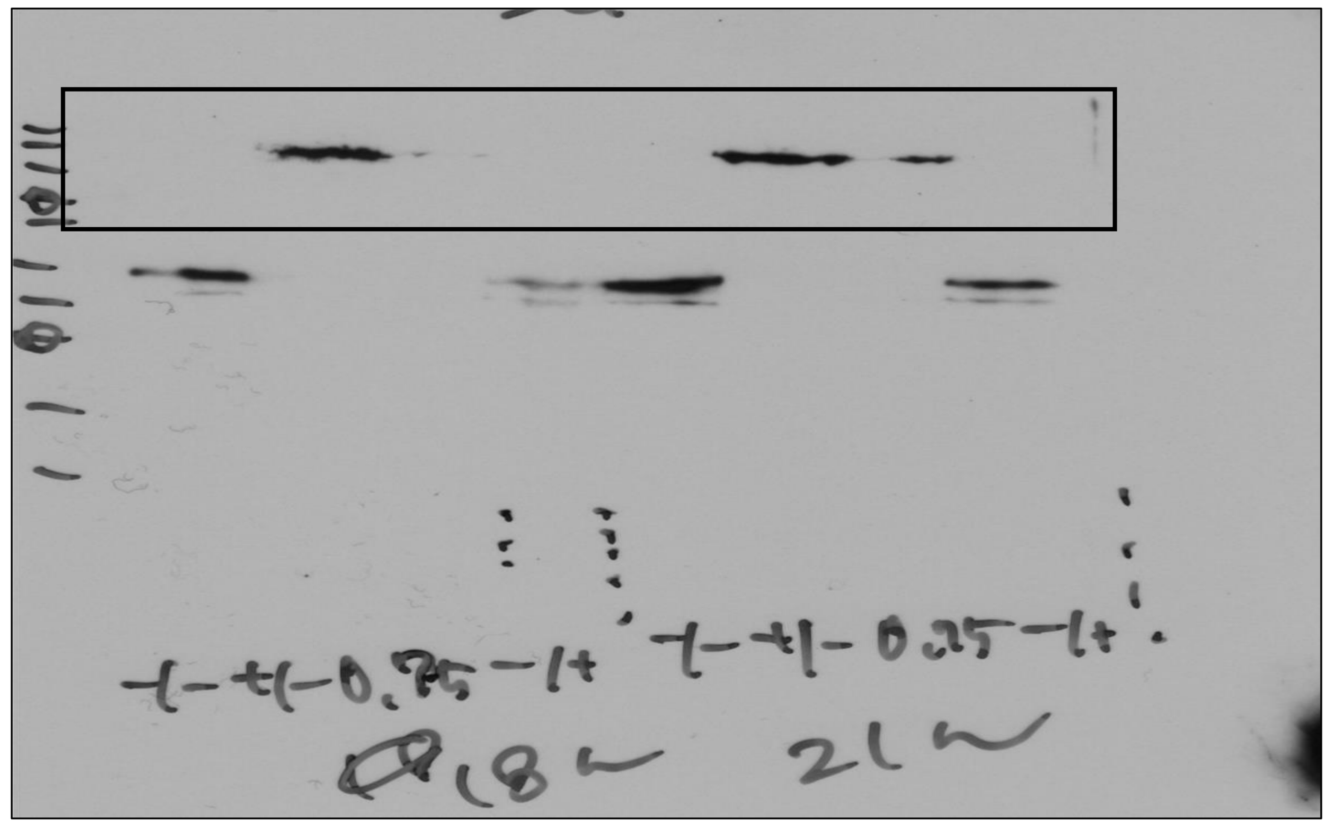

Blot: ICP5

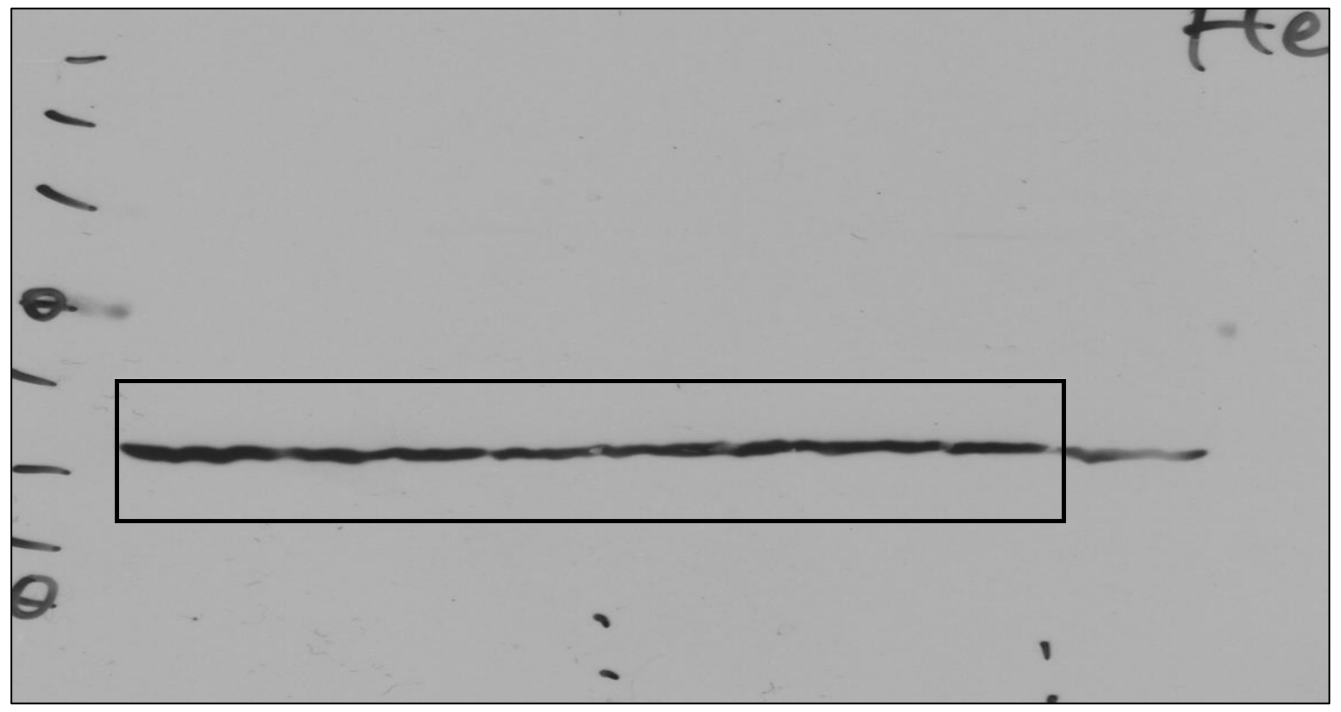

Blot: β-Actin

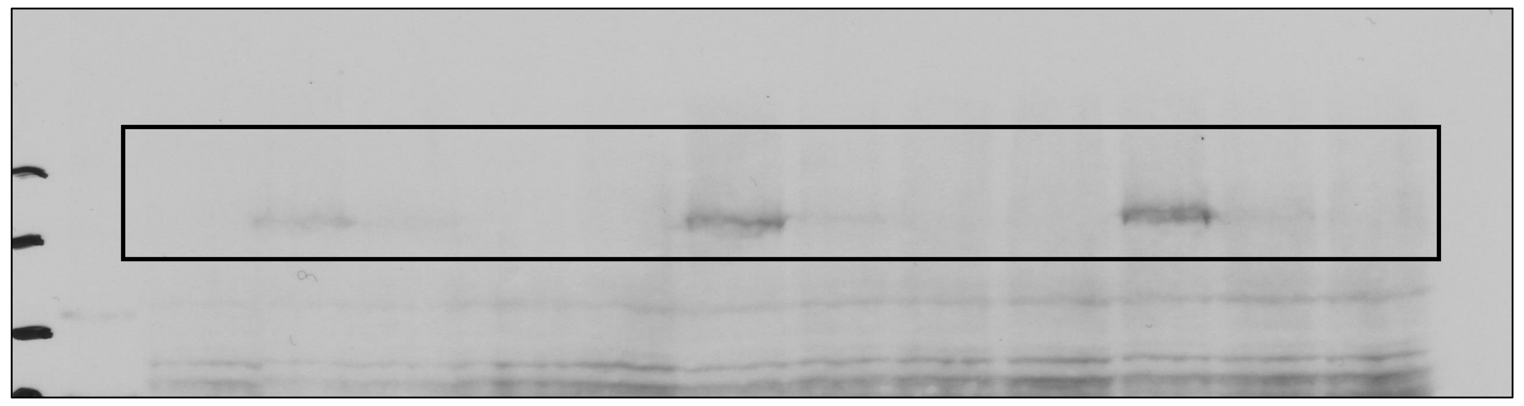

Blot: ICP5

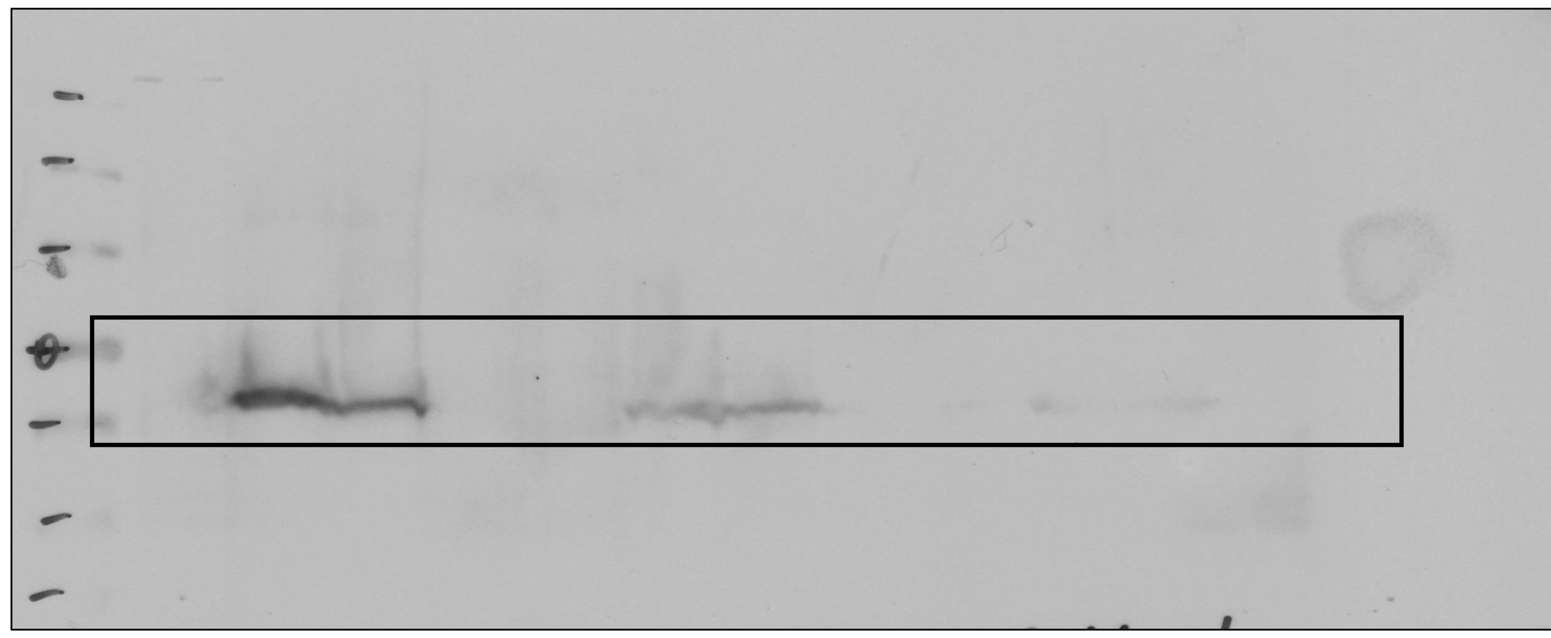

Blot: UL42

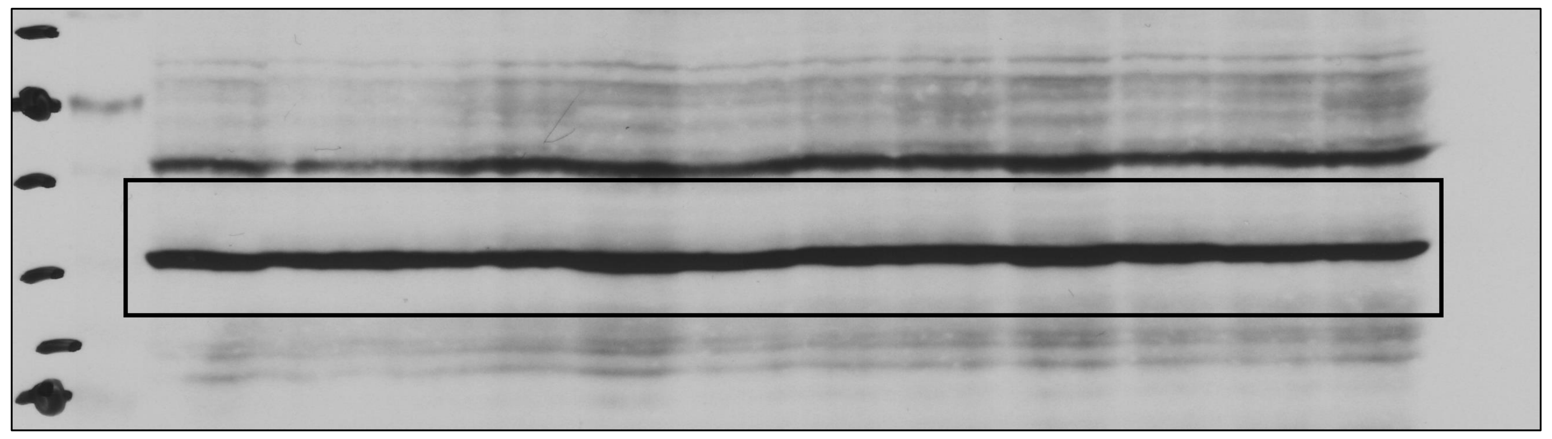

Blot: β-Actin

Supplementary Fig. S3 (original data of Figure 4a 4b, and 4e)

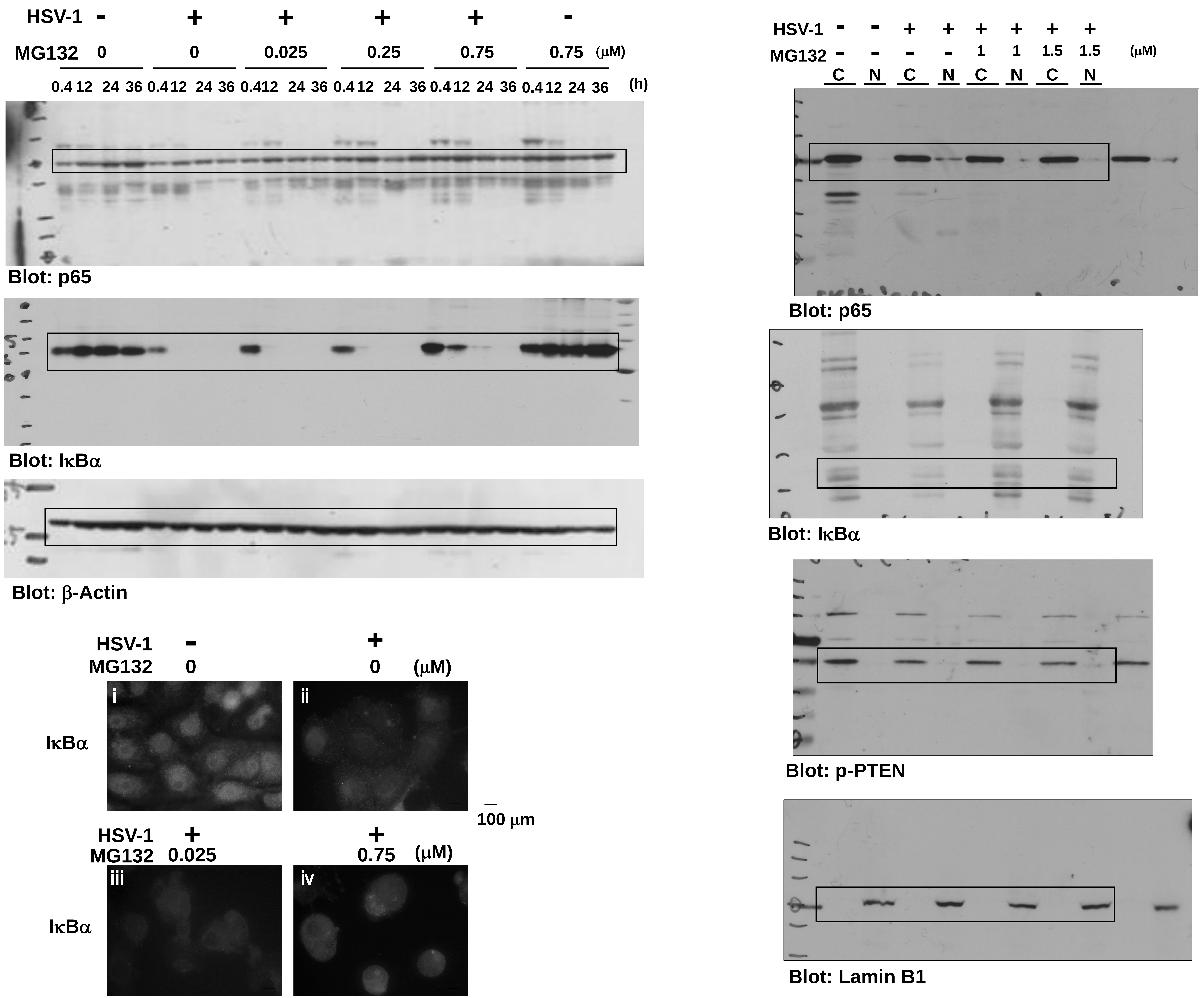

Supplementary Fig. S4 (original data of Figure 5)

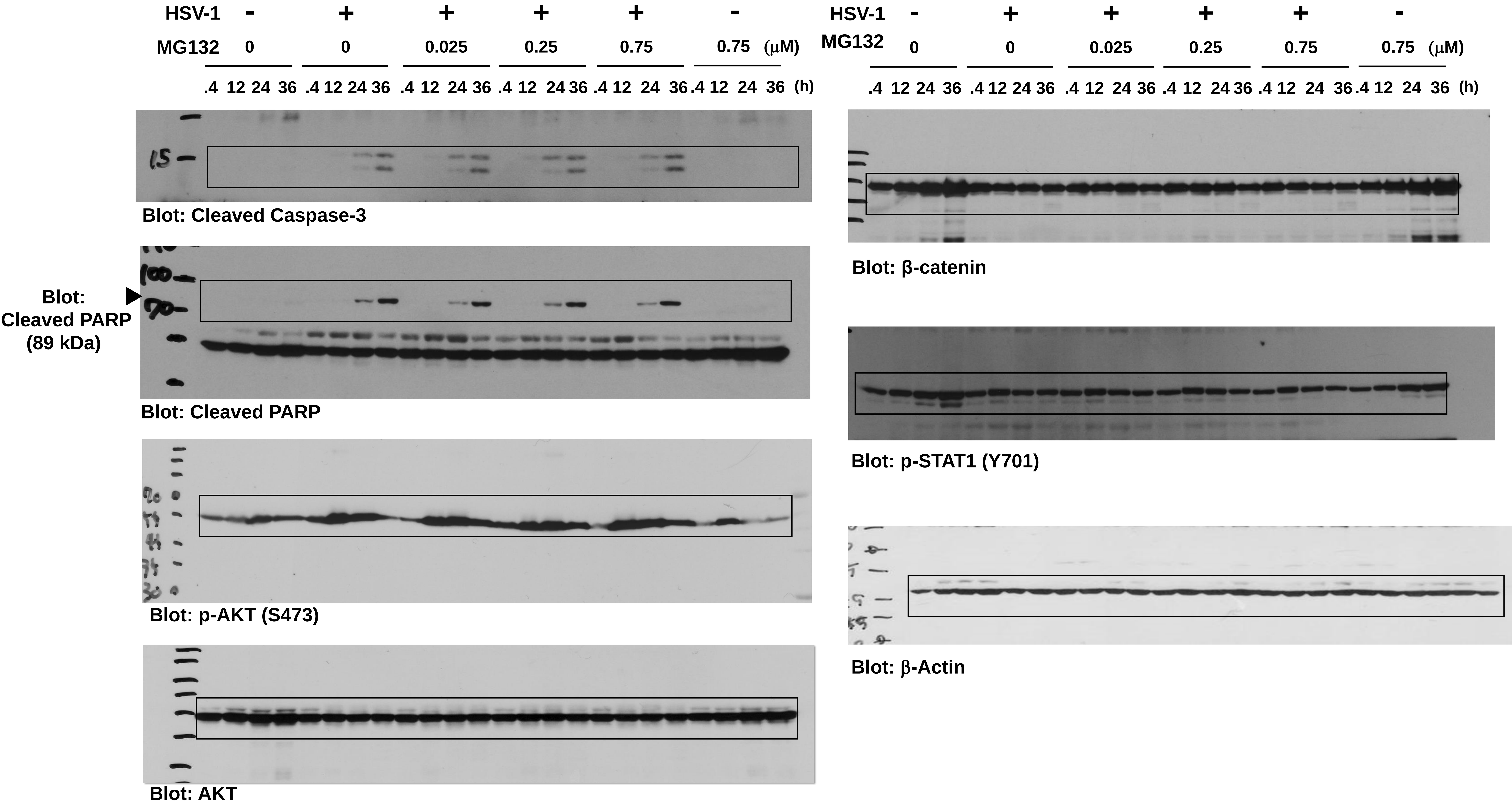

Supplementary Fig. S5 (original data of Figure 6)

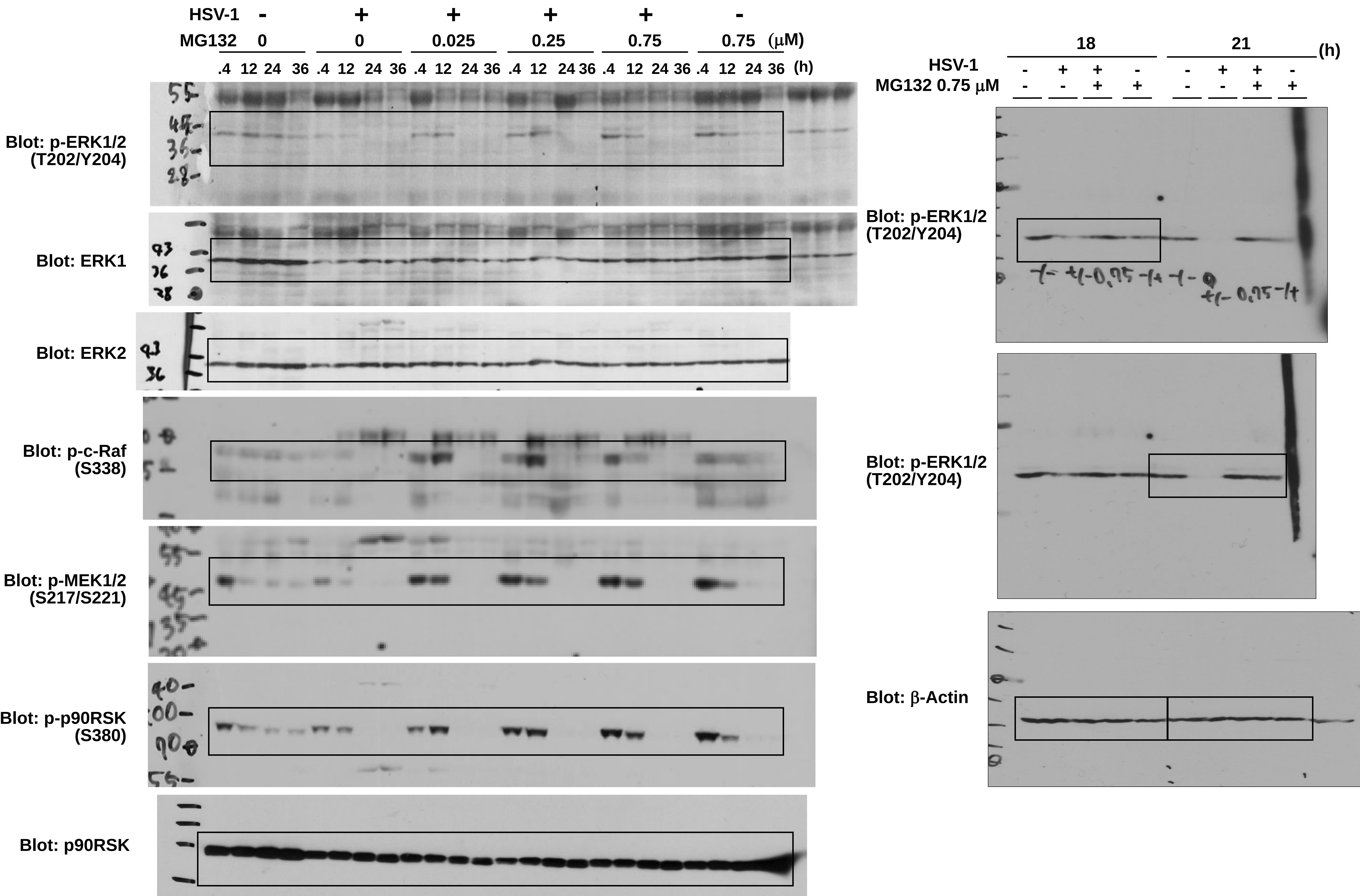

Supplementary Fig. S6 (original data of Figure 8a, 8c and 8g)

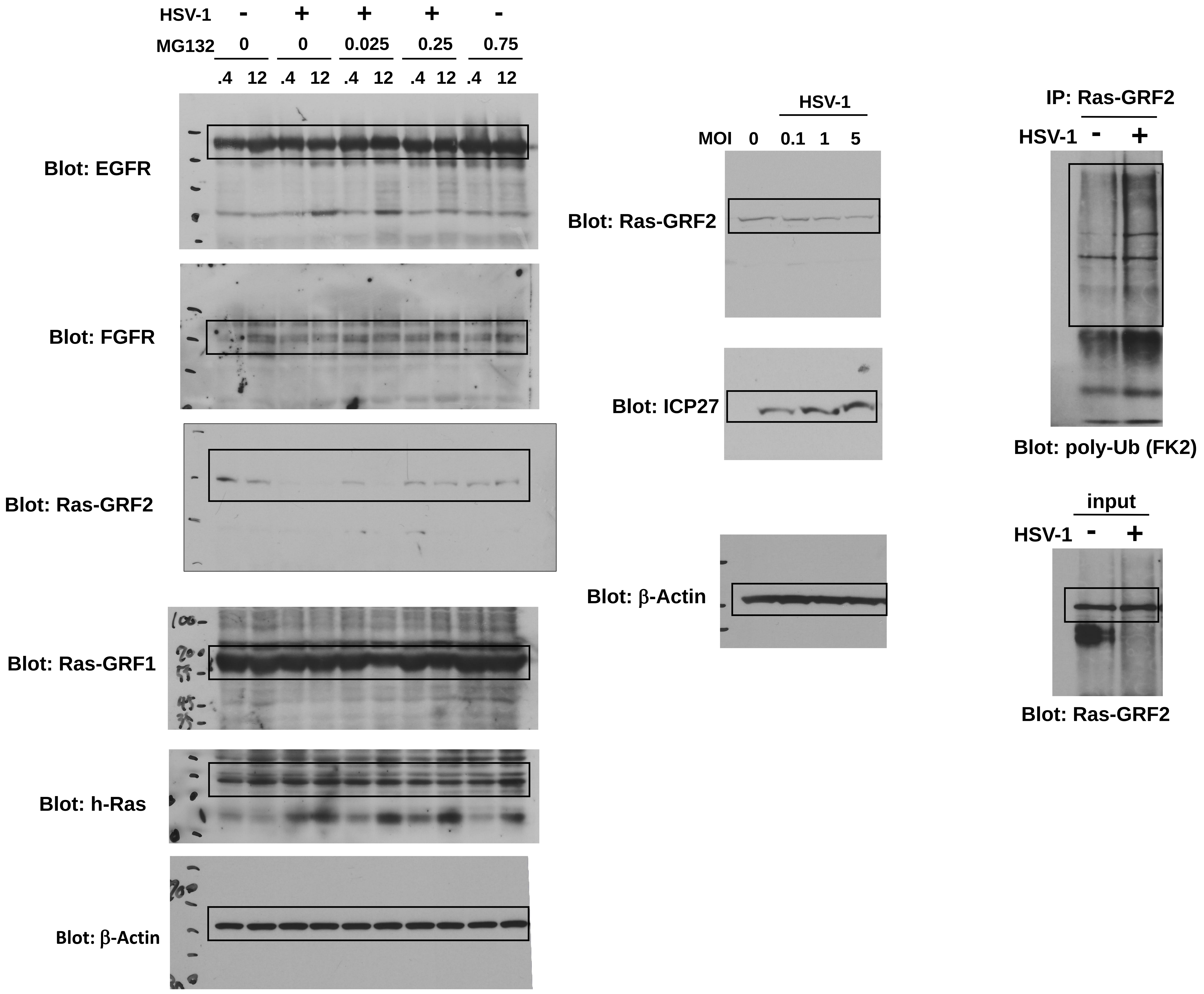

Supplement: Supplementary file 1 — Supplementary Information. [file 41598_2020_63438_MOESM1_ESM.pdf]
